# Vitamin B_12_ as a carrier of peptide nucleic acid (PNA) into bacterial cells

**DOI:** 10.1038/s41598-017-08032-8

**Published:** 2017-08-09

**Authors:** Marcin Równicki, Monika Wojciechowska, Aleksandra J. Wierzba, Jakub Czarnecki, Dariusz Bartosik, Dorota Gryko, Joanna Trylska

**Affiliations:** 1 College of Inter-Faculty Individual Studies in Mathematics and Natural Sciences, Banacha 2c, 02-097 Warsaw, Poland; 2 Centre of New Technologies, University of Warsaw, Banacha 2c, 02-097 Warsaw, Poland; 3 Institute of Organic Chemistry, Polish Academy of Sciences, M. Kasprzaka 44/52, 01-224 Warsaw, Poland; 4 Department of Bacterial Genetics, Institute of Microbiology, Faculty of Biology, University of Warsaw, Miecznikowa 1, 02-096 Warsaw, Poland

## Abstract

Short modified oligonucleotides targeted at bacterial DNA or RNA could serve as antibacterial agents provided that they are efficiently taken up by bacterial cells. However, the uptake of such oligonucleotides is hindered by the bacterial cell wall. To overcome this problem, oligomers have been attached to cell-penetrating peptides, but the efficiency of delivery remains poor. Thus, we have investigated the ability of vitamin B_12_ to transport peptide nucleic acid (PNA) oligomers into cells of *Escherichia coli* and *Salmonella* Typhimurium. Vitamin B_12_ was covalently linked to a PNA oligomer targeted at the mRNA of a reporter gene expressing Red Fluorescent Protein. Cu-catalyzed 1,3-dipolar cycloaddition was employed for the synthesis of PNA-vitamin B_12_ conjugates; namely the vitamin B_12_ azide was reacted with PNA possessing the terminal alkyne group. Different types of linkers and spacers between vitamin B_12_ and PNA were tested, including a disulfide bond. We found that vitamin B_12_ transports antisense PNA into *E. coli* cells more efficiently than the most widely used cell-penetrating peptide (KFF)_3_K. We also determined that the structure of the linker impacts the antisense effect. The results of this study provide the foundation for developing vitamin B_12_ as a carrier of PNA oligonucleotides into bacterial cells.

## Introduction

**Figure 1:**
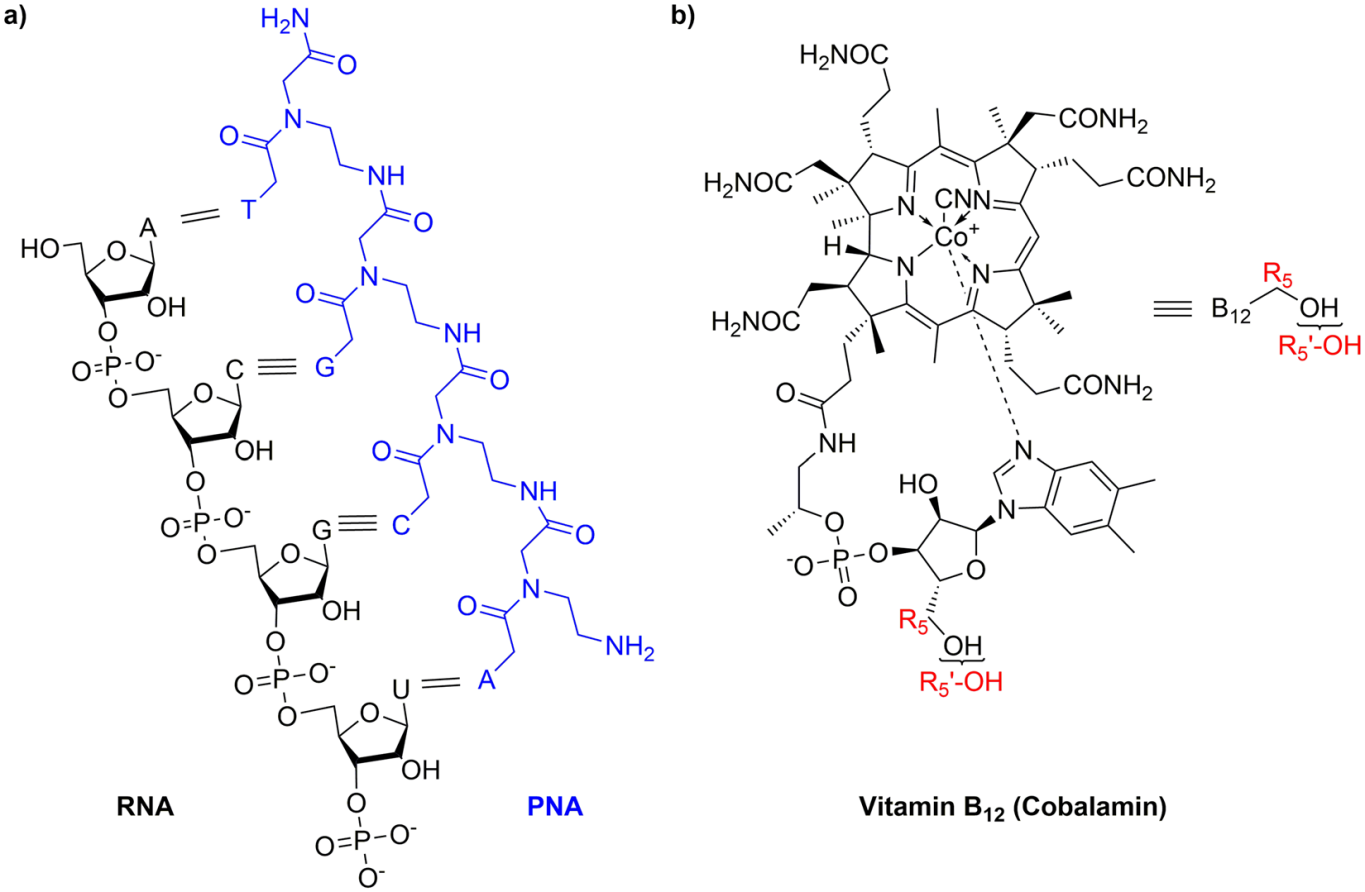
(**a**) Chemical structure of a PNA oligomer and its complementary pairing scheme with natural RNA. (**b**) Vitamin B_12_ in the form of cyanocobalamin used in this study. R_5_ refers to the fifth carbon of the ribose moiety, while R_5_′-OH stands for the primary hydroxyl group according to the nomenclature of cobalamin^4^.

The rapid development and spread of antimicrobial resistance motivates the search for new antibiotics. In principle, the use of modified sequence-specific oligonucleotides as steric blockers of bacterial RNA or DNA seems a promising strategy. Traditional antisense strategies use short oligonucleotides that hybridize with complementary mRNA sequences through Watson-Crick base pairing and block translation^1, 2^. The advantage of this approach is that oligomer sequences can be rapidly redesigned if bacterial resistance arises due to mutation of the target. Since natural oligonucleotides are rapidly degraded in the intracellular environment, chemically-modified oligonucleotides such as Peptide Nucleic Acids (PNA)^3^ have been used (Fig. 1a).

PNA oligomers containing a pseudo-peptide instead of a sugar-phosphate backbone show improved nuclease resistance, lower toxicity and increased affinity of hybridization with natural nucleic acids^5^. PNAs have been successfully tested as antimicrobials in a variety of bacterial species^2, 6, 7^. To inhibit bacterial growth, the mRNAs of several essential genes have been targeted, including *acpP*, the gene for the acyl carrier protein*, gyrA* encoding DNA gyrase subunit A, and *murA* and *fabI*, genes involved in cell wall and fatty acid biosynthesis, respectively^3, 8, 9^.In a separate approach, functional fragments of both 23S and 16S rRNA have been verified as targets for antisense PNAs^10–13^.

The most serious drawback, which currently precludes the use of this approach, is poor delivery of the oligonucleotides into bacterial cells. The bacterial cell wall prevents the efficient uptake of short oligonucleotides from the environment^14^ and non-invasive delivery of PNAs is extremely difficult. To overcome this problem, chemical conjugation of PNA to a variety of cell-penetrating peptides has been tested^7, 15, 16^. The most commonly used conjugate of PNA with the peptide (KFF)_3_K was delivered with high efficiency *in vitro*. However, the activity of (KFF)_3_K-PNA conjugates was dramatically decreased in the presence of serum in eukaryotic cells^17^. Furthermore, as a cationic peptide (KFF)_3_K possesses hemolytic activity at the very low concentration of 40 µg/ml (for comparison, the antibiotic polymyxin B is not hemolytic at 1500 µg/ml)^18^. In addition, (KFF)_3_K initiates histamine release in some mammalian cells, which leads to the development of an inflammatory response and causes pruritis^6^. For these reasons (KFF)_3_K is not a good delivery agent for neutral oligonucleotides and its future medical application is doubtful. A variety of other membrane-penetrating peptides have been tested *in vitro*, including TAT^15^ and (RXR)_4_XB (X – 6-aminohexanoic acid, B – β-alanine)^2^, oncocin^19^, and others^20–22^ but with variable results. All of these peptides are cationic and amphipathic^23^, and their efficiency as PNA transporters is still far from perfect^24^. Moreover, we recently observed that conjugation with (KFF)_3_K decreased the ability of PNA to efficiently hybridize with an RNA hairpin even though the peptide is positively charged and thus attracted by the negatively charged RNA^13^. Also, conjugation of (KFF)_3_K with PNA (targeted at a functional site in 23S rRNA) decreased the level of inhibition of protein production in an *E. coli* cell-free transcription/translation system as compared to PNA alone. Therefore, there is still a pressing need for an effective carrier system to deliver PNAs to bacterial cells.

Vitamin B_12_ (cobalamin) is a natural organometallic molecule (Fig. 1b)^25^. It is an essential nutrient cofactor in mammalian metabolism^26^. Vitamin B_12_ cannot be synthesized within the human body so must be included in the diet^27^, which makes this molecule an attractive and easy-to-administer candidate as a drug carrier. In recent studies, vitamin B_12_ has been used as a delivery vehicle in mammalian cells^25^ and applied to increase the bioavailability of different therapeutics including proteins^28^ and anti-cancer drugs^29^. Most aerobic bacteria require vitamin B_12_ for growth, but only a few species are able to produce it^30^. A variety of microorganisms are capable of its uptake^30^, especially members of the *Enterobacteriaceae* family, *Bacillus subtilis* and Group A streptococci^31, 32^. *Escherichia coli (E. coli)* and *Salmonella enterica* serovar Typhimurium (*S*. Typhimurium) cells actively transport vitamin B_12_ using a cascade of membrane proteins^33^. The import of vitamin B_12_ into these cells involves a rapid energy-independent phase in which the molecule associates with the receptor BtuB in the outer membrane. This initial stage is followed by a slower energy-dependent process involving other membrane proteins. Furthermore, *Salmonella* species possess a second independent system for the passage of vitamin B_12_ across the outer membrane^34^. The presence of fairly well characterized vitamin B_12_ uptake mechanisms in bacteria makes it an attractive candidate for a carrier. To exploit the natural uptake properties of vitamin B_12_, the molecule has to be modified and conjugated to PNA oligomers. It was shown that conjugation at certain positions dramatically alters the binding properties of vitamin B_12_ in mammalian cells^35^. To successfully use the vitamin B_12_ pathway in the transport of PNA, the conjugate must still be recognized by the B_12_ uptake mechanism, and the PNA has to interact with its target and cause the desired effect.

We have investigated the ability of the non-peptidic carrier vitamin B_12_ to transport PNA oligomers into *E. coli* and *S*. typhimurium cells. Vitamin B_12_ was conjugated to a PNA oligomer targeted at the mRNA of the reporter gene *mrfp1* ^36^, expressing Red Fluorescent Protein (RFP), in *E. coli* and *S*. Typhimurium cells. Decreases in RFP fluorescence were monitored to detect any PNA activity within cells treated with these compounds. Since the uptake of conjugates may depend on the linker, we tested different linker types and spacer lengths, including a cleavable disulfide bond linker.

## Results and Discussion

### System to monitor inhibition of *mrfp1* mRNA translation

**Figure 2:**
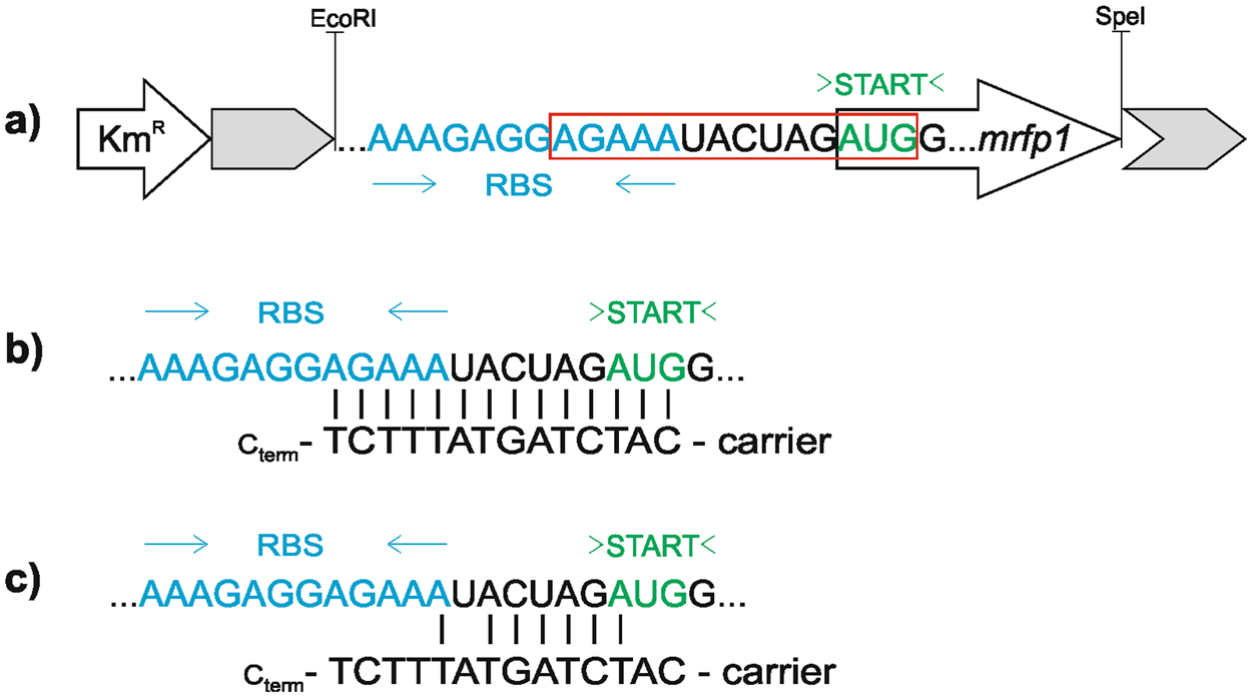
(**a**) Schematic representation and construction strategy of *mrfp1* expression plasmid pBBR(rfp). The red rectangle surrounds the mRNA target site for the PNA sequence. Km^R^ refers to the kanamycin resistance marker; gray arrows represent the *lacZ* gene encoding β-galactosidase, disrupted by the insert (RBS and *mrfp1)*. The plasmid contains two other structural elements: rep and mob which were not included in the figure. (**b**) Anti-*mrfp1* PNA sequence. c) Control scrambled PNA. The carrier is either (KFF)_3_K or vitamin B_12_.

To provide a convenient system for comparative studies of the effect of antisense PNA in *E. coli* and *S*. Typhimurium, we constructed pBBR(rfp), an RFP reporter vector optimized for expression in *Enterobacteriaceae* (Fig. 2a). Gene *mrfp1* was chosen as the reporter because any antisense effect could be readily assessed by examining red fluorescence of the cells (Figure S1). The anti-*mrfp1* PNA was designed to target the region of the mRNA overlapping the translation start codon, which was shown to be sensitive to antisense inhibition^37^, plus part of the ribosome binding site (RBS) B0034 (http://parts.igem.org/Part:BBa_B0034) (Fig. 2b). This RBS is recognized as strong and highly efficient (http://parts.igem.org/Part:BBa_K1017202). To ensure that the PNA sequence was specific for the *mrfp1* mRNA, we examined off-target gene complementarity using online sequence analysis tools - GenoList^38^ and RiboScanner^10^. We also verified the physicochemical properties of the PNA oligomer, which could affect its solubility (PNA Tool http://pnabio.com/).

### Synthesis of vitamin B_12_- PNA conjugates

**Figure 3:**
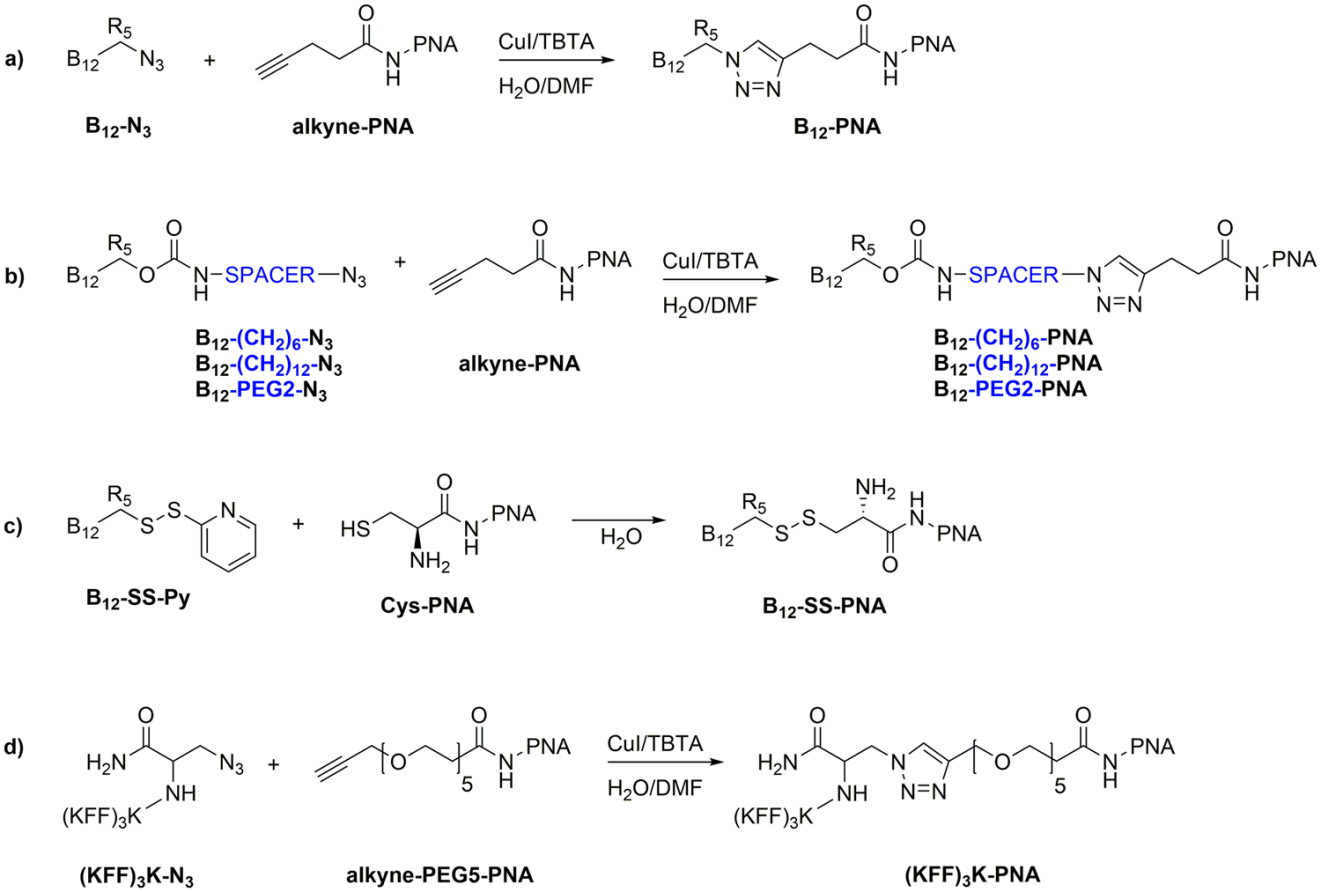
Synthesis of vitamin B_12_ and (KFF)_3_K conjugates with PNA. The spacer is marked in blue.

We designed and synthesized a series of conjugates to be delivered into bacterial cells, by attaching the PNA oligomer to vitamin B_12_, incorporating both cleavable and non-cleavable linkers between the molecules. 1,3-dipolar cycloaddition was used to prepare four such conjugates (Fig. 3a-b). In one conjugate, alkyne-PNA was directly coupled to vitamin B_12_ possessing an azide moiety at 5′ position – B_12_-N_3_ (Fig. 3a)^39^, while in the other three, a spacer (alkyl- or PEG-type) was incorporated into the vitamin B_12_ structure via the carbamate bond^40, 41^, and this was then coupled to the alkyne-PNA (Fig. 3b). To introduce a cleavable linker, Cys-PNA was reacted with the B_12_-SS-Py derivative to produce a conjugate containing a disulfide bridge (Fig. 3c)^42^. As controls, we also synthesized PNA conjugates (anti*-mrfp1* and scrambled sequences) with the (KFF)_3_K peptide. The azide derivative of this peptide ((KFF)_3_K-N_3_) was attached to the alkyne-PEG5-PNA via 1,3-dipolar cycloaddition (Fig. 3d)^43^. See Methods for detailed synthesis procedures.

In order to achieve the desired inhibitory effect in cells, the conjugates need to be stable. Thus we examined the stability of the vitamin B_12_-PNA conjugates *in vitro* in the bacterial Davis Minimal Broth^44^ medium and fetal bovine serum. The resulting HPLC chromatograms did not show any appreciable differences before and after incubation so all conjugates were considered stable in the presence of biological media.

### Verification of the system

**Figure 4:**
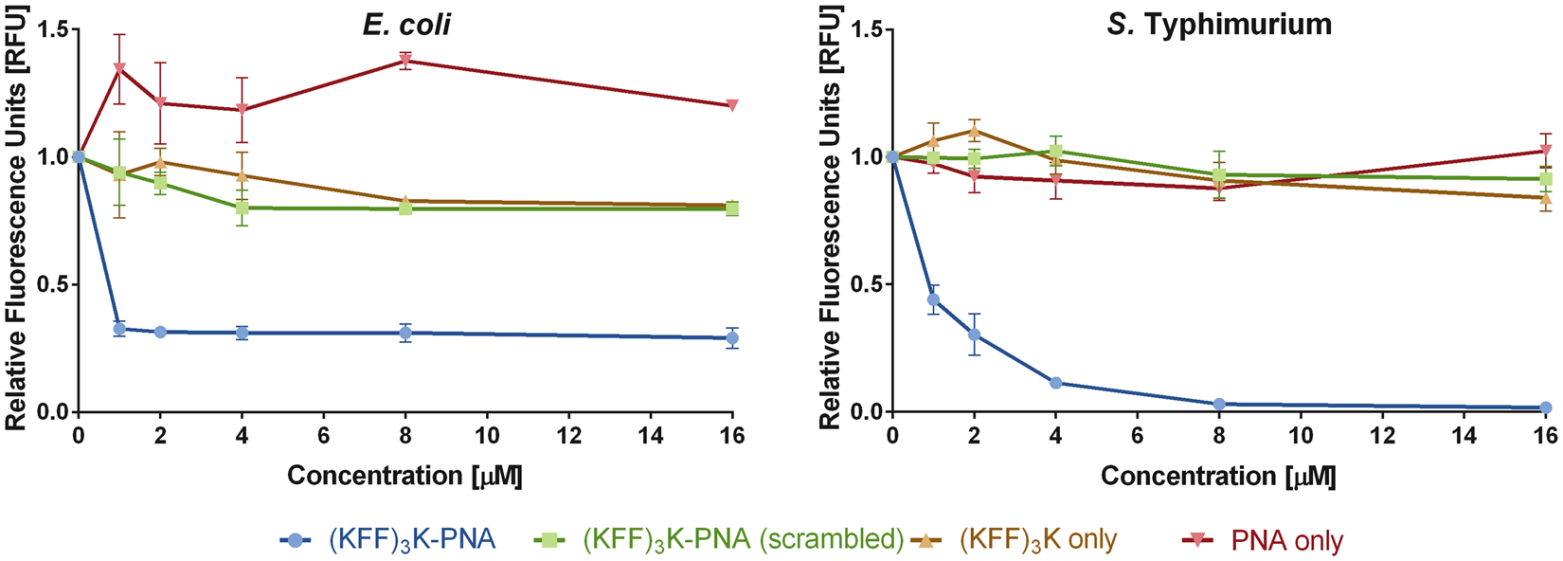
Inhibition of RFP synthesis after treating cells with (KFF)_3_K-PNA, (KFF)_3_K-PNA (scrambled), PNA anti-m*rfp1* or free (KFF)_3_K. For the sequence of the PNA targeted at the mRNA transcript encoding RFP, see Fig. 2. The mean values from three independent experiments are plotted, with error bars indicating the standard error. The fluorescence intensity given in relative fluorescence units [RFU] correlates well with the cellular RFP level (see Methods). Data for all tested concentrations are shown in Table S1. The differences between RFU obtained for (KFF)_3_K-PNA and each of the other compounds are highly significant with P ≤ 0.001.

To evaluate the potential of antisense PNA to inhibit translation of the *mrfp1* mRNA transcript, we cultured bacteria in the presence of anti*-mrfp1* PNA conjugated to the (KFF)_3_K peptide ((KFF)_3_K-PNA, Fig. 3d). This cell-penetrating peptide is frequently employed to transport PNA into bacterial cells^8^, so it was used as a control. As shown in Fig. 4, we observed a significant decrease in red fluorescence in bacteria grown in the presence of (KFF)_3_K-PNA (relative to untreated cells). At all tested concentrations of this conjugate, the level of RFP was reduced by about 70% in *E. coli*. However, in *S*. Typhimurium the production of RFP decreased in a dose-dependent manner following (KFF)_3_K-PNA treatment. In addition, the antisense effect of (KFF)_3_K-PNA on RFP production in *S*. Typhimurium reached almost 100% at concentrations of ≥8 µM. This variation between two members of the *Enterobacteriaceae* probably results from phenotypic and genotypic differences^45, 46^, such as structural diversity in the core regions of the lipopolysaccharides^47^ which may affect PNA transport. To confirm that the decrease in RFP expression was caused by the anti*-mrfp1* PNA, we tested a scrambled PNA sequence (Fig. 2c). RFP production was unchanged following treatment with this altered PNA (Fig. 4). Furthermore, there was no inhibition of *mrfp1* expression when either free (KFF)_3_K or the PNA sequence without the carrier peptide were applied (Fig. 4). Therefore, the inhibitory effect that we observed was dependent on the PNA sequence delivered in a conjugate.

Interestingly, *E. coli* cells treated with PNA only exhibit higher fluorescence intensity than cells treated with (KFF)_3_K-PNA(scrambled) or (KFF)_3_K (Fig. 4). We hypothesize that this effect may be due to electrostatically-driven interactions of (KFF)_3_K with RFP that could influence the detected level of fluorescence. The (KFF)_3_K peptide is positively charged (with a total net charge of +4e) and the RFP protein is negatively charged (total net charge of −5e and pI of 6.1^48^). On the other hand PNA is neutral so is not attracted by the RFP protein.

To rule out unspecific toxicity of the compounds we monitored their effect on bacterial growth. The measurements of OD_600_ after overnight incubation of bacterial cells with the above compounds (at concentrations required to deliver PNA to cells) did not indicate bacterial growth inhibition and thus antibacterial activity. The minimal inhibitory concentration for free (KFF)_3_K is >20 μM in both *E. coli* and *S*. Typhimurium^10, 13^. Surprisingly, after treatment with PNA only, we observed a slightly higher level of OD_600_ than for untreated cells but this observation corroborates with the observed RFU (Fig. 4).

### Inhibition of RFP synthesis by vitamin B_12_ conjugates with anti-*mrfp1* PNA

**Figure 5:**
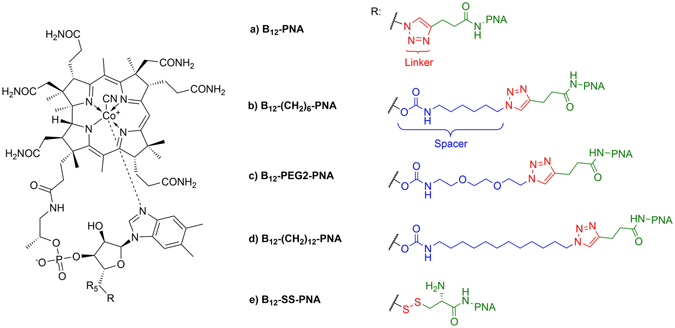
Structures and naming of vitamin B_12_-PNA conjugates. The carrier is at the PNA N-terminus and PNA C-terminus contains Lys.

We next investigated the ability of five different vitamin B_12_ derivatives conjugated to the anti*-mrfp1* PNA to transport this PNA into Gram-negative bacterial cells and inhibit RFP synthesis. The tested compounds shown in Fig. 4 vary in the way in which vitamin B_12_ was connected to the PNA. In four of the compounds, vitamin B_12_ and the PNA were conjugated via the triazole ring, which is stable in the biological environment and, most importantly, in the broth used for culturing bacteria (Fig. 5a–d)^49^. These compounds differed in the length of the spacer. In conjugate B_12_-SS-PNA (Fig. 5e), vitamin B_12_ was connected to PNA via a disulfide bond that can be reduced by glutathione (GSH), an antioxidant molecule widely distributed in bacteria. The level of antisense inhibition of RFP translation by these conjugates was determined by measuring changes in the red fluorescence of *E. coli* and *S*. Typhimurium cells following overnight treatment (see Methods).

**Figure 6:**
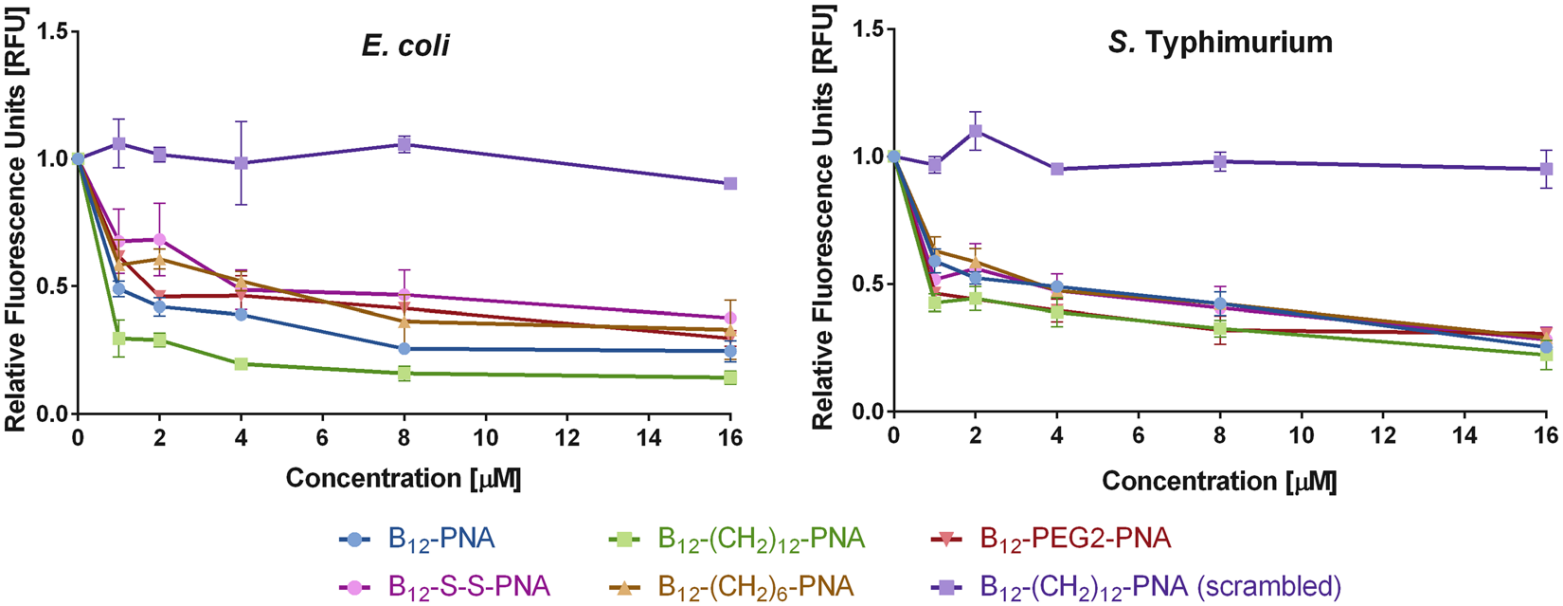
Relative fluorescence measured in cells after treatment with conjugates of vitamin B_12_-PNA targeted at the mRNA encoding RFP. Data for all tested concentrations are shown in Table S2. The differences between RFU of B_12_-(CH_2_)_12_-PNA(scrambled) and the rest of tested conjugates are highly significant with P ≤ 0.001. In addition, for *E. coli*: the difference between B_12_-(CH_2_)_12_-PNA and B_12_-(CH_2_)_6_-PNA is significant with P ≤ 0.01 and the difference between B_12_-(CH_2_)_12_-PNA and B_12_-PEG2-PNA is marginally significant (P ≤ 0.05). The differences between other tested conjugates are not significant (P > 0.05).

Antisense effects were detected with all of the vitamin B_12_-PNA conjugates tested on *E. coli* and *S*. Typhimurium (Fig. 6). Moreover, we observed that the structure and length of the linker and spacer affected the delivery of PNA. The conjugates that most effectively inhibited RFP synthesis had either no spacer (B_12_-PNA) or the longest spacer (B_12_-(CH_2_)_12_-PNA), especially in *E. coli* cells. The two other compounds with the triazole ring as a linker, i.e. B_12_-(CH_2_)_6_-PNA and B_12_-PEG2-PNA, caused a lesser antisense effect. Vitamin B_12_ with the degradable linker B_12_-SS-PNA inhibited *mrfp1* gene expression with the lowest efficiency. This was anticipated because disulfide bonds are generally not stable in the cytosolic compartments of bacteria or eukaryotic cells, due to their chemically reducing nature^50^.

Similar results were obtained for both *E. coli* and *S*. Typhimurium, although in the latter bacterium, the inhibition of RFP production by all compounds was less effective (~75% at most). In addition, the nature of the linkers and spacers present caused no significant difference in the inhibitory activity of the vitamin B_12_-PNA conjugates in *S*. Typhimurium. This different effect in these two bacteria might be due to differences in the structure of the cell wall as well as vitamin B_12_ requirements for growth and the membrane transport systems involved in uptake^34, 51^.

As a control for these experiments, we used the scrambled PNA (Fig. 2c) attached to vitamin B_12_ via –(CH_2_)_12_ (Fig. 5d), i.e. the linker that inhibited RFP production most effectively when conjugated to the complementary PNA. Free vitamin B_12_ was used as a second control. Neither B_12_–(CH_2_)_12_-PNA(scrambled) (Fig. 6) nor free vitamin B_12_ (data not shown) had any inhibitory effect on *mrfp1* expression.

In addition, the vitamin B_12_-PNA conjugates did not affect bacterial growth. There was no decrease in OD_600_ upon overnight treatment with the conjugates (up to the tested 16 μM concentrations). Free vitamin B_12_ has no antibacterial activity at concentrations up to 100 μg/ml^52^. However, it has some growth-promoting properties^53^, which resulted in a slight increase of OD_600_ while increasing the concentration of vitamin B_12_.

In *E. coli* for all vitamin B_12_-PNA conjugates, we observed a concentration-dependent decrease in red fluorescence (Fig. 6). In contrast, with the (KFF)_3_K-PNA conjugate, we saw an initial drop in fluorescence at 0.125 µM, followed by a steady level of inhibition at higher concentrations (Fig. 4 and Table S1). A similar inhibitory effect was observed when all conjugates were applied at 2 µM. In *E. coli*, at concentrations between 4 and 16 µM, the decrease in fluorescence produced by the most effective vitamin B_12_-(CH_2_)_12_-PNA constructs was greater than for (KFF)_3_K-PNA (Figure S2).

The conjugate of PNA with the (KFF)_3_K peptide produced a different effect in *S*. Typhimurium (Fig. 4). In addition, the (KFF)_3_K-PNA and vitamin B_12_-PNA conjugates showed comparable dose-dependent inhibition of RFP fluorescence in this bacterium (Figs 4 and 6). A similar efficiency of RFP inhibition was produced by (KFF)_3_K-PNA and B_12_-(CH_2_)_12_-PNA at 1 µM (Figure S2). Overall, the delivery efficiency of PNA to *S*. Typhimurium cells was better for PNA conjugated with (KFF)_3_K than vitamin B_12_, when applied at concentrations of between 2 and 16 µM.

Overall, in *E. coli* for the conjugates with the longest B_12_-(CH_2_)_12_-PNA and shortest B_12_-PNA linker we observed that PNA transport efficiency by vitamin B_12_ is slightly better than by the (KFF)_3_K peptide but for *S*. Typhimurium the effect was the opposite. One reason could be that the uptake occurs only up to certain concentrations of the conjugate that are too low for the PNA to achieve its antisense effect in cells (and these maximal concentration of vitamin B_12_ uptake is lower in *S*. Typhimurium than *E. coli*). Also, we have recently observed that conjugation of PNA with the (KFF)_3_K peptide hinders PNA hybridization efficiency with a complementary RNA strand^13^. To determine if the attachment of vitamin B_12_ affects hybridization of a PNA oligomer with complementary RNA, we performed polyacrylamide gel electrophoresis (PAGE) experiments. We used the best working conjugates and an RNA oligomer with the _5_′AGGAGAAAUACUAGAUGGCU_3_′ sequence corresponding to the targeted mRNA transcript (with the fragment complementary to PNA underlined). With the secondary structure prediction programs (MFold^54^, RNAfold^55^, and Sfold^56^) we verified that this RNA sequence most probably does not acquire any secondary structure and does not self-interact. The mRNA fragment was incubated in water solution containing either PNA or the B_12_-PNA and B_12_-(CH_2_)_12_-PNA conjugates (in a 1:1 ratio) and assayed by PAGE in non-denaturing conditions. We found that after adding PNA to RNA (Figure S3, lane 2) the band from free RNA disappeared confirming that free PNA binds to RNA. However, after adding PNA conjugated to vitamin B_12_ to RNA (Figure S3, lanes 3 and 4), the band from the unbound RNA was still present. This means that although the band from the complex is visible, the attachment of vitamin B_12_ to PNA somehow interferes with the formation of the complex. Thus similar as we previously observed for the (KFF)_3_K carrier^13^, vitamin B_12_ may also decrease the ability of PNA to bind complementary RNA.

In summary, we have demonstrated that vitamin B_12_ can deliver PNA oligomers into *E. coli* and *S*. Typhimurium cells. However, attaching of vitamin B_12_ to PNA may influence PNA hybridization with a complementary fragment of the targeted mRNA transcript encoding RFP. Even though the attachment of vitamin B_12_ does not favor hybridization of PNA with mRNA the antisense effect of PNA is still visible. The vitamin B_12_-PNA conjugates were stable in bacterial media and serum and did not inhibit bacterial growth. In the future we plan to investigate whether vitamin B_12_ can also be used to transport PNA into the cells of Gram-positive bacteria.

## Methods

### Reagents and conditions

Commercial reagents and solvents were used as received from the supplier. Fmoc-XAL PEG PS resin for PNA synthesis was obtained from Merck and Fmoc/Bhoc-protected PNA monomers from Panagene. Nα-Fmoc protected L-amino acids were obtained from Novabiochem (Fmoc-Lys(Boc)-OH) and Sigma-Aldrich (Fmoc-Phe-OH, Fmoc-Cys(Trt)-OH, Fmoc-β-azido-Ala-OH). Rink-amide resin (TentaGel S RAM resin) for peptide synthesis was obtained from Sigma-Aldrich. All reactions were monitored using Reverse Phase-HPLC (RP-HPLC) techniques. Preparative chromatography was performed using C18 reversed-phase silica gel 90 Å (Sigma-Aldrich) with redistilled water and HPLC grade MeCN as eluents. The following conditions were used for HPLC: column – Eurospher II 100–5 C18, 250 mm × 4.6 mm with a precolumn, or Kromasil C18, 5 µm, 250 mm × 4.0 mm; pressure – 10 MPa; flow rate – 1 mL/min; room temperature; detection – UV/vis at wavelengths (λ) of 361 and 267 nm. HPLC methods, molecular masses and yields are given in Table 1. Details of the ^1^H and ^13^C NMR spectra are presented in Section S1.

**Table 1:** Retention times (t_R_) and molecular masses of the synthesized conjugates.

Nr	Conjugate	HPLC t_R_ [min]^a^	HPLC method	Molecular mass [g/mol]		Yields^c^ [%]
				Calculated	Found^b^	
1.	B_12_-PNA	15.2	0–50%/30 min	5339.4	5340.5	81.1
2.	B_12_-(CH_2_)_6_-PNA	17.1	10–45%/30 min	5482.6	5482.7	79.6
4.	B_12_-PEG_2_-PNA	15.5	0–50%/30 min	5514.6	5516.0	67.6
5.	B_12_-(CH_2_)_12_-PNA	20.0	0–50%/30 min	5566.8	5566.5	73.5
6.	B_12_-SS-PNA	14.7	0–50%/30 min	5353.3	5352.0	53.1
7.	B_12_-(CH_2_)_12_-PNA (scrambled)	20.6	0–50%/30 min	5690.2	5567.2	56.8
8.	(KFF_3_)K-PNA	20.6	0–50%/30 min	5691.2	5690.0	79.6
9.	(KFF)_3_K-PNA (scrambled)	20.9	0–50%/30 min	5690.1	5690.2	57.0

^a^The product was analyzed by analytical RP-HPLC; ^b^data obtained from Q-TOF Premier ^c^isolated yield on 1 µmol scale.

### Preparation of vitamin B_12_ derivatives at the 5′ position

B_12_-PEG2-N_3_ and B_12_-(CH_2_)_12_-N_3_ were synthesized in the following way. Vitamin B_12_ (100 mg, 75 μmol) was dissolved in 2.5 mL of dry DMSO at 40 °C in an argon atmosphere. Solid CDT (50 mg, 300 μmol) was added and the solution stirred under argon. When full consumption of the substrate (monitored by RP-HPLC) had occurred (usually after 1.5 h) heating was removed and 100 µL of aminoazide NH_2_-PEG2-N_3_ or B_12_-(CH_2_)_6_-N_3_ was added in one portion, followed by 20 µL of NEt_3_. The resulting solution was stirred overnight and then it was poured into 50 mL of AcOEt and centrifuged. The pelleted precipitate was then washed with Et_2_O (2 × 15 mL). After drying in air, the precipitate was dissolved in water and purified by RP column chromatography with a mixture of MeCN and H_2_O as the eluent. The experimental details and the complete characterization of B_12_-(CH_2_)_12_-N_3_ and B_12_-PEG2-N_3_ derivatives are presented in Section S1, and Figures S4 and S5, respectively. Vitamin B_12_ derivatives B_12_-(CH_2_)_12_-N_3_, B_12_-PEG2-N_3_ and B_12_-(CH_2_)_6_-N_3_ were synthesized according to previously described procedures^39, 41, 42^. The obtained spectral data matched that in the literature. The NH_2_-(CH_2_)_12_-N_3_ linker was synthesized according to the procedure described in^57^ and the NH_2_-PEG2-N_3_ and NH_2_-(CH_2_)_6_-N_3_ linkers were synthesized according to^41^. Again, the obtained spectral data matched that in the literature.

### Synthesis of PNA oligomers

Cys-PNA was synthesized according to the procedure of Wierzba *et al*. (2016)^42^. PNA oligomers (alkyne-PNA, alkyne-PEG5-PNA, Cys-PNA, alkyne-PEG5-PNA scrambled) were synthesized manually using Fmoc chemistry at 10 µmol scale with a 2.5-fold molar excess of the Fmoc/Bhoc-protected monomers and 3-fold molar excess of the Fmoc-protected Lys, pentynoic acid, alkyne-PEG5-acid and polyethylene glycol-polystyrene resin (Fmoc-XAL PEG PS resin, amine groups loading of 190 μmol/g; this resin has a linker which yields a C-terminal amide upon TFA cleavage of PNA). In all syntheses Lys was the first monomer attached to resin. Monomers were activated by treatment with a 2-(1H-7-azabenzotriazole-1-yl)-1,1,3,3-tetramethyluronium hexafluorophosphate (HATU), N-methylmorpholine (NMM) and 2,6-lutidine (0.7:1:1.5) mixture using DMF/NMP (1:1, v/v) solution, and coupled for 40 min as active derivatives. A double coupling was performed. Fmoc deprotection was accomplished using 20% piperidine in DMF (2 × 2 min). After synthesis of the PNA backbone and removal of the N-terminal Fmoc, the pentynoic acid or alkyne-PEG5-acid were attached to the N-terminus. Acids were assembled as active derivatives in 3-fold molar excess by the use of HATU with the addition of HOAt and collidine (1:1:2), using the DMF/NMP (1:1, v/v) solution-coupling method for 2 h. Fmoc deprotection of amino acids was accomplished using 20% piperidine in DMF for 2 cycles (5 and 15 min). Removal of the protecting group and cleavage of PNA from the resin was performed by treatment with a TFA/triisopropylsilane/m-cresol (95:2.5:2.5; v/v/v) mixture for 60 min. The obtained crude oligomers were lyophilized and subsequently purified by RP-HPLC.

### Synthesis of (KFF)_3_K-N_3_

Azido-peptide was synthesized by manual solid-phase peptide synthesis (SPPS) using the standard Fmoc/t-Bu chemistry on a 100 µmol scale with a 3-fold molar excess of the Fmoc-protected amino acids and Rink-amide resin (TentaGel S RAM resin, amine groups loading of 240 μmol/g; this resin has a linker which yields a C-terminal amide upon TFA cleavage of the peptide). Fmoc-protected amino acids were assembled as active derivatives in a 3-fold molar excess by the use of HATU with the addition of 1-hydroxy-7-azabenzotriazole (HOAt) and collidine (1:1:2), using the DMF/NMP (1:1, v/v) solution-coupling method for 2 h. Fmoc deprotection was accomplished using 20% piperidine in DMF for 2 cycles (5 and 15 min). Removal of the protecting group (Boc from Lys) and cleavage of peptide from the resin was performed by treatment with a TFA/triisopropylsilane/m-cresol (95:2.5:2.5; v/v/v) mixture for 60 min. The obtained crude peptide was lyophilized and subsequently purified by RP-HPLC.

### Synthesis of PNA conjugates with carriers: vitamin B_12_ and (KFF)_3_K

The B_12_-SS-PNA conjugate was prepared by the method reported in ref. 42. Other conjugates were synthesized using copper-catalyzed azide-alkyne cycloaddition according to the procedures described in refs 39, 43. CuI (1.0 mg, 5 μmol) and TBTA (5.0 mg, 10 μmol) were dissolved in DMF/H_2_O (0.5 mL, 1:1 v/v) and stirred for 20 min. The respective azide-B12 or azide-peptide (3 µmol) and the respective alkyne-PNA (1 µmol) were then added and the reaction mixtures were stirred overnight. The mixtures were centrifuged to remove the catalyst and the solutions, containing the crude products, were then purified by RP-HPLC. Table 1 gives the experimental details and the physicochemical properties of the final compounds. According to HPLC analyses all reactions proceeded with conversion > 99%. HPLC and MS analysis of the B_12_-PNA and (KFF)_3_K-PNA products gave m/z values in accordance with their calculated molecular masses. Yields of the final isolated products were in the range 53–81% on 1 µmol synthesis scale. The purity, determined by RP-HPLC (267 nm), was ≥ 98% for all the conjugates. Mass spectra and RP-HPLC chromatograms are shown in Section S2, Figures S6–S13. To monitor the stability, the vitamin B_12_-PNA conjugates were added at 50 µM concentrations to either Davis Minimal Broth^44^ medium or fetal bovine serum. After overnight incubation at 37 °C with shaking the RP-HPLC analyses were performed under the same conditions as described above.

### Bacterial strains and growth conditions

*E. coli* TG1^58^ was used for plasmid construction. For triparental mating, *E. coli* DH5α carried the helper plasmid pRK2013^59^ and *E. coli* S17-1^60^ was the *mrfp1* plasmid donor. *E. coli* K-12 MG1655^61^, and *Salmonella enterica* subsp*. enterica* serovar Typhimurium LT2-R (rifampicin resistant mutant of wild-type *S*. Typhimurium LT2^62^) were used in PNA delivery experiments. To prepare inocula, all strains were grown overnight in lysogeny broth (LB) at 37 °C with shaking. To monitor inhibition of *mrfp1* gene expression the analyzed strains were grown in Davis Minimal Broth at 37 °C with shaking. Cultures were supplemented with appropriate antibiotics to prevent plasmid loss.

### Construction of the RFP vector

Plasmid pSB3K3-RFP contains the *mrfp1* gene with rbs_B0034, an efficient ribosomal binding site (http://parts.igem.org/Part:BBa_K1017202). Plasmid pBBR1MCS-2 is a cloning vector optimized for *Enterobacteriaceae* ^63^ and functional in the majority of Gram-negative bacteria. Plasmid pBBR(rfp), which constitutively expresses RFP, was constructed by ligating an EcoRI-SpeI restriction fragment containing the *mrfp1* gene and rbs_B0034 from pSB3K3-RFP to pBBR1MCS-2 digested by the same endonucleases (Figure S14). Appropriate kits were used for the isolation of plasmid DNA from bacterial cells and its purification after enzymatic reactions (EURx, Gdańsk, Poland). Restriction enzymes and ligase were purchased from Thermo Scientific™. Common DNA manipulation methods were performed as described by Sambrook and Russell^58^.

### Introduction of plasmid pBBR(rfp) into bacterial cells

#### *E. coli* transformation

Chemical competent *E. coli* TG1 cells were prepared using the *E. coli* Transformer kit (A&A Biotechnology) following the supplied protocol. A 100 µL aliquot of frozen competent cells (stored at −80 °C) was thawed on ice, then 20 µL of ligation reaction were added and the cells gently mixed. Following incubation on ice for 45 min, the transformation mixture was placed in a thermoblock at 37 °C for 10 min. The mixture was then cooled on ice for 2 min and 100 μl were spread on an LB agar plate containing 50 µg/ml kanamycin. Plates were incubated overnight at 37 °C to permit growth of transformants. The expression of RFP was verified by the detection of red fluorecence when colonies were examined on a UV transilluminator.

### Triparental mating with *S*. Typhimurium

Triparental mating^64^ was used to mobilize plasmid pBBR(rfp) into the rifampicin resistant mutant of *S*. Typhimurium LT2 (LT2-R). Overnight cultures of *E. coli* S17-1 carrying mobilizable vector pBBR(rfp) (donor), *E. coli* DH5α carrying plasmid pRK2013 (helper) and *S*. Typhimurium LT2-R (recipient) were centrifuged. The pelleted cells were washed once in LB to remove antibiotics and then resuspended in LB. The donor, helper and recipient strains were then mixed in a volume ratio of 1:1:2, respectively. 100 µL of this cell mixture were spread on a plate of LB agar and incubated overnight at 37 °C. The bacteria were then washed off the plate and serially diluted 1:10, 1:100, and 1:1000 in LB. 100 µl samples of the undiluted cell suspension and each of the dilutions were plated on LB agar containing rifampicin (50 µg/ml) (selectable marker for the recipient strain) and kanamycin (50 µg/ml) and incubated overnight at 37 °C. Transconjugant colonies were identified by the detection of red fluorescence as described above (Figure S15). Restriction digest analysis of isolated plasmid DNA verified the presence of pBBR(rfp) in 5–10 fluorescent clones (Figure S14). To confirm that transconjugants were *S*. Typhimurium, 16 S rDNA Restriction Fragment Length Polymorphism (RFLP) analysis was performed.

### Amplification of 16S rDNA genes and RFLP analysis

For DNA preparation, small loopfuls of *S*. Typhimurium or *E. coli* cells were suspended in lysis buffer (0.25% SDS, 50 mM NaOH) and heated at 99 °C for 10 min. The lysate was then diluted with distilled water and this was used as the template DNA in PCRs. Universal bacterial 16 S rRNA primers were included in the reactions: forward primer 27 F (5′- AGAGTTTGATCMTGGCTCAG -3′)^65^ corresponding to positions 8 to 27 in *E. coli* rRNA and reverse primer 1492R^66^ corresponding to positions from 1491 to 1509 (5′- GGTTACCTTGTTACGACTT-3′). The PCR reactions (25 µL) had the following composition: 1 µL of template DNA, 1 µL of each 16 S rRNA primer (0.5 µM), 12.5 µL Thermo Scientific™ DreamTaq™ (LifeTechnologies) DNA Polymerase mixture, 9.5 µL ddH_2_0. The following thermocycle was employed in the PCR: initial denaturation at 95 °C for 180 s, followed by 20 cycles of 95 °C for 50 s, 53 °C for 50 s, and 72 °C for 90 s; then 15 cycles of 95 °C for 30 s, 46 °C for 30 s and 72 °C for 90 s, finishing with a final extension step at 72 °C for 10 min.

### 16s rDNA Restriction Fragment Length Polymorphism

Amplified 16 S rDNA PCR products were purified and then digested separately with the restriction enzyme SalI, which was predicted to produce different restriction fragment patterns for the two species (Figure 16 caption). A 16 S rDNA fragment amplified from *E. coli* was cleaved with the same enzyme as a control. The digested DNA fragments were analyzed by agarose gel (0.8%) electrophoresis (60 min at 100 V) and the gel was stained with ethidium bromide for 20 min before viewing on a UV transilluminator (Figure S16).

### Determination of the level of red fluorescence

The effect of the different PNA conjugates and controls on the red fluorescence of bacterial cells was determined using a standard microdilution method^67^ in concentration range 0–16 µM. Cultures grown to exponential phase in Davis Minimal Broth were diluted to ~5 × 10^5^ CFU/ml. These cell suspensions were added to wells of sterile 96-well plates containing different concentrations of the tested compounds. Following overnight incubation at 37 °C with shaking, the plates were vigorously shaken (double orbital) for 10 sec and then the cell density (OD_600_) and fluorescence (λ excitation – 584 nm; λ emission – 610 nm) were measured using a plate reader (Microplate Reader Biotek Synergy H1MFDG). The cell suspensions were also examined by light and fluorescence microscopy using a Nikon Eclipse Ni-U microscope (Figure S1).

### Fluorescence Data analysis

To obtain relative fluorescence values (RFU), the background media noise was subtracted from each data point for both cell density (OD_600_) and fluorescence. Then the background-adjusted fluorescence data were divided by the background-adjusted OD_600_ and the data were normalized to the untreated cells. This value is proportional to the amount of RFP per cell, which correlates with the PNA activity. Free vitamin B_12_ was not taken into account because it shows negligible fluorescence emission. To evaluate statistical significance the two-way ANOVA was used. A probability value of P ≤ 0.05 was considered indicative of a statistical significance.

### PAGE analysis

Polyacrylamide gel electrophoresis (PAGE) was performed using the Mini-PROTEAN Tetra Cell system (Bio-Rad, Poland). Sample mixtures (200 pmol each) were transferred onto the gel using a solution of 40% glycerol in water (loading buffer). For 5 µL samples, 1 µL of loading buffer was added, then samples were electrophoresed under 60 V for 3 h on a 15% non-denaturing polyacrylamide gel, using 1x TBE as a running buffer (89 mM Tris-base, 89 mM borate, 2 mM EDTA, pH 8.3). Gels were stained by Stains-All (Sigma-Aldrich) and imaged using a Gel Doc XR + System (Bio-Rad).

## Electronic supplementary material

Supporting Information
