# Supplementary material for: Vitamin B12 as a carrier of peptide nucleic acid (PNA) into bacterial cells
Source: Sci Rep. 2017 Aug 9;7:7644. doi: 10.1038/s41598-017-08032-8 (PMC5550456; doi:10.1038/s41598-017-08032-8)
Supplement: Supplementary file 1 — Supporting Information [file 41598_2017_8032_MOESM1_ESM.pdf]

**Supporting Information for**

# Vitamin $B_{12}$ as a carrier of peptide nucleic acid (PNA) into bacterial cells

Marcin Równicki[1,2], Monika Wojciechowska[2], Aleksandra J. Wierzba[3], Jakub Czarnecki[4], Dariusz Bartosik[4], Dorota Gryko[3,*], Joanna Trylska[2,*]

[1] College of Inter-Faculty Individual Studies in Mathematics and Natural Sciences, Banacha 2c, 02-097 Warsaw, Poland
[2] Centre of New Technologies University of Warsaw, Banacha 2c, 02-097 Warsaw, Poland
[3] Institute of Organic Chemistry, Polish Academy of Sciences, M. Kasprzaka 44/52, 01-224 Warsaw, Poland
[4] Department of Bacterial Genetics, Institute of Microbiology, Faculty of Biology, University of Warsaw, Miecznikowa 1, 02-096 Warsaw, Poland

* dorota.gryko@icho.edu.pl and joanna@cent.uw.edu.pl

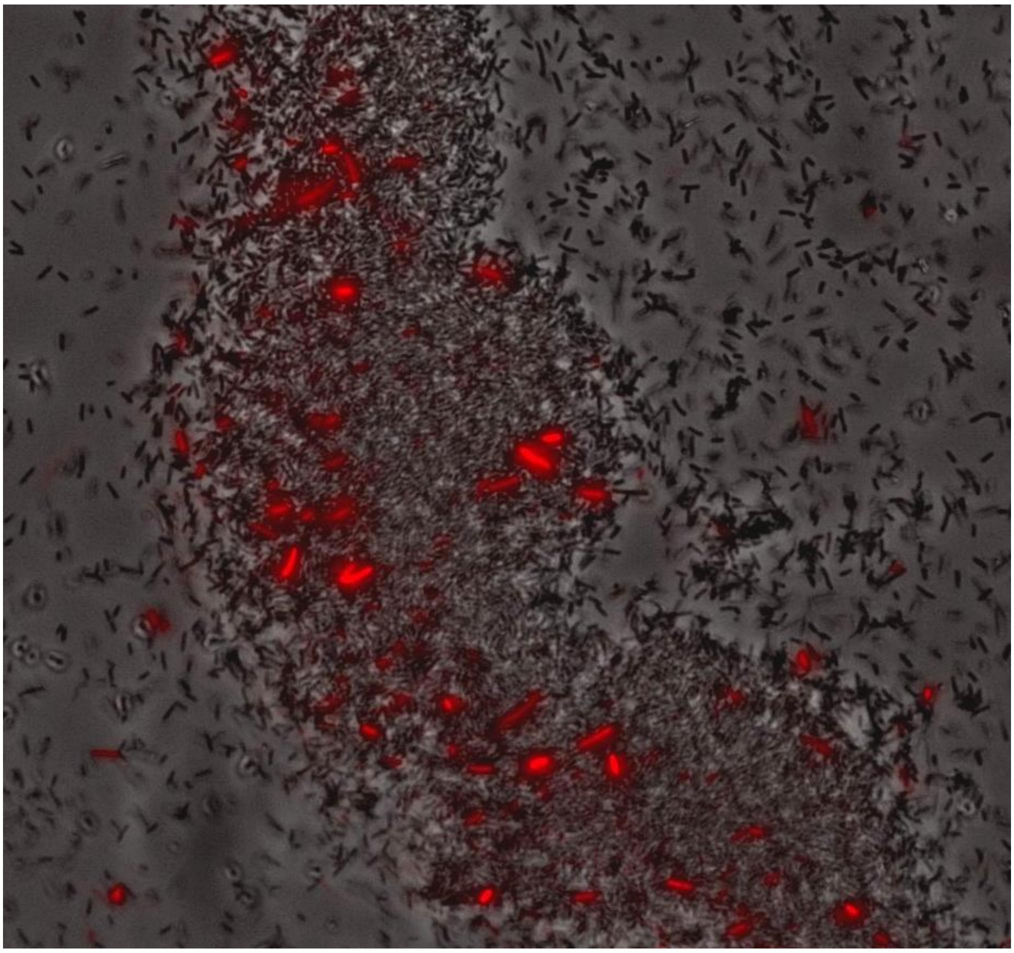

**Figure S1.** Two superimposed pictures: 1st from light microscopy (showing the general amount of *E. coli* cells in the microscopic slide) and from fluorescence microscopy showing strong inhibition of red cellular fluorescence after overnight treatment with 16 µM $B_{12}$-$(CH_2)_{12}$-PNA targeted at mRNA encoding RFP.

## *E. coli*

| | Relative Fluorescence Units [RFU] | | | | | | | | | | | |
|---|---|---|---|---|---|---|---|---|---|---|---|---|
| µM | $(KFF)_3K$-PNA | | | $(KFF)_3K$-PNA (scrambled) | | | $(KFF)_3K$ only | | | PNA only | | |
| **0** | 1.00 | 1.00 | 1.00 | 1.00 | 1.00 | 1.00 | 1.00 | 1.00 | 1.00 | 1.00 | 1.00 | 1.00 |
| **0.125** | 0.42 | 0.29 | 0.30 | 0.91 | 1.11 | 0.81 | 0.89 | 1.07 | 0.93 | 1.31 | 1.09 | 1.24 |
| **0.25** | 0.46 | 0.29 | 0.40 | 1.21 | 0.86 | 0.89 | 0.87 | 0.82 | 0.72 | 1.59 | 1.45 | 1.47 |
| **0.5** | 0.48 | 0.29 | 0.34 | 1.21 | 0.87 | 0.97 | 0.97 | 0.72 | 1.04 | 1.19 | 1.11 | 1.19 |
| **1** | 0.38 | 0.28 | 0.32 | 1.17 | 0.93 | 0.72 | 1.26 | 0.70 | 0.83 | 1.48 | 1.07 | 1.48 |
| **2** | 0.35 | 0.28 | 0.31 | 0.90 | 0.82 | 0.97 | 1.06 | 0.88 | 1.00 | 1.37 | 0.89 | 1.37 |
| **4** | 0.28 | 0.29 | 0.36 | 0.78 | 0.93 | 0.69 | 1.11 | 0.86 | 0.81 | 1.31 | 0.93 | 1.31 |
| **8** | 0.28 | 0.27 | 0.38 | 0.76 | 0.79 | 0.84 | 0.84 | 0.81 | 0.83 | 1.41 | 1.31 | 1.41 |
| **16** | 0.37 | 0.26 | 0.24 | 0.75 | 0.80 | 0.84 | 0.85 | 0.79 | 0.79 | 1.22 | 1.16 | 1.22 |

## *S.* Typhimurium

| | Relative Fluorescence Units [RFU] | | | | | | | | | | | |
|---|---|---|---|---|---|---|---|---|---|---|---|---|
| µM | $(KFF)_3K$-PNA | | | $(KFF)_3K$-PNA (scrambled) | | | $(KFF)_3K$ only | | | PNA only | | |
| **0** | 1.00 | 1.00 | 1.00 | 1.00 | 1.00 | 1.00 | 1.00 | 1.00 | 1.00 | 1.00 | 1.00 | 1.00 |
| **0.125** | 0.93 | 0.86 | 0.86 | 0.86 | 0.67 | 1.26 | 1.14 | 1.02 | 1.15 | 1.08 | 1.11 | 1.25 |
| **0.25** | 0.77 | 0.82 | 0.78 | 1.15 | 0.82 | 0.96 | 1.19 | 1.16 | 1.02 | 1.26 | 1.08 | 0.90 |
| **0.5** | 0.67 | 0.62 | 0.62 | 0.94 | 0.83 | 1.14 | 0.90 | 1.41 | 0.96 | 0.73 | 0.83 | 0.92 |
| **1** | 0.33 | 0.47 | 0.52 | 0.98 | 1.04 | 0.97 | 1.09 | 0.93 | 1.17 | 0.90 | 1.02 | 1.00 |
| **2** | 0.14 | 0.38 | 0.39 | 1.02 | 1.04 | 0.92 | 1.06 | 1.19 | 1.06 | 0.87 | 1.05 | 0.85 |
| **4** | 0.07 | 0.13 | 0.14 | 0.91 | 1.06 | 1.10 | 0.99 | 0.89 | 1.08 | 0.84 | 0.83 | 1.05 |
| **8** | 0.03 | 0.03 | 0.03 | 0.99 | 0.75 | 1.05 | 0.80 | 0.88 | 1.04 | 0.79 | 0.95 | 0.89 |
| **16** | 0.02 | 0.02 | 0.01 | 0.91 | 0.83 | 1.00 | 0.86 | 0.92 | 0.74 | 1.13 | 1.04 | 0.90 |

**Table S1.** Extended data for Figure 4 of the main text. 1.00 stands for normalized fluorescence units for the untreated cells. Colors of the compounds are as in the legend of Figure 4.

## *E. coli*

| | Relative Fluorescence Units [RFU] | | | | | | | | | | | | | | | | | |
|---|---|---|---|---|---|---|---|---|---|---|---|---|---|---|---|---|---|---|
| µM | $B_{12}$-PNA | | | $B_{12}$-$(CH_2)_{12}$-PNA | | | $B_{12}$-$(CH_2)_6$-PNA | | | $B_{12}$-S-S-PNA | | | $B_{12}$-PEG2-PNA | | | $B_{12}$-$(CH_2)_{12}$-PNA (scrambled) | | |
| **0*** | 1.00 | 1.00 | 1.00 | 1.00 | 1.00 | 1.00 | 1.00 | 1.00 | 1.00 | 1.00 | 1.00 | 1.00 | 1.00 | 1.00 | 1.00 | 1.00 | 1.00 | 1.00 |
| **0.125** | 0.60 | 0.65 | 0.73 | 0.54 | 0.73 | 0.76 | 1.16 | 1.03 | 0.88 | 0.89 | 0.72 | 0.98 | 0.76 | 0.55 | 0.87 | 1.28 | 1.22 | 1.13 |
| **0.25** | 0.52 | 0.57 | 0.63 | 0.46 | 0.48 | 0.61 | 0.80 | 0.61 | 1.00 | 1.00 | 0.79 | 0.69 | 0.92 | 1.01 | 0.51 | 1.14 | 1.30 | 0.80 |
| **0.5** | 0.42 | 0.48 | 0.57 | 0.31 | 0.31 | 0.46 | 0.77 | 0.90 | 0.93 | 0.97 | 0.73 | 0.65 | 0.65 | 0.63 | 0.50 | 1.28 | 1.22 | 1.13 |
| **1** | 0.43 | 0.51 | 0.53 | 0.21 | 0.24 | 0.44 | 0.40 | 0.74 | 0.61 | 0.92 | 0.61 | 0.50 | 0.64 | 0.62 | 0.59 | 0.96 | 0.97 | 1.25 |
| **2** | 0.35 | 0.44 | 0.47 | 0.24 | 0.30 | 0.33 | 0.63 | 0.66 | 0.53 | 0.95 | 0.64 | 0.46 | 0.46 | 0.49 | 0.43 | 0.96 | 1.04 | 1.05 |
| **4** | 0.36 | 0.40 | 0.41 | 0.18 | 0.20 | 0.21 | 0.51 | 0.46 | 0.59 | 0.43 | 0.39 | 0.64 | 0.31 | 0.53 | 0.55 | 1.31 | 0.82 | 0.82 |
| **8** | 0.23 | 0.27 | 0.27 | 0.11 | 0.16 | 0.21 | 0.27 | 0.25 | 0.57 | 0.65 | 0.32 | 0.43 | 0.43 | 0.42 | 0.39 | 1.00 | 1.06 | 1.11 |
| **16** | 0.17 | 0.26 | 0.31 | 0.10 | 0.14 | 0.19 | 0.20 | 0.23 | 0.56 | 0.40 | 0.35 | 0.38 | 0.34 | 0.24 | 0.31 | 0.89 | 0.90 | 0.92 |

## *S.* Typhimurium

| | Relative Fluorescence Units [RFU] | | | | | | | | | | | | | | | | | |
|---|---|---|---|---|---|---|---|---|---|---|---|---|---|---|---|---|---|---|
| µM | $B_{12}$-PNA | | | $B_{12}$-$(CH_2)_{12}$-PNA | | | $B_{12}$-$(CH_2)_6$-PNA | | | $B_{12}$-S-S-PNA | | | $B_{12}$-PEG2-PNA | | | $B_{12}$-$(CH_2)_{12}$-PNA (scrambled) | | |
| **0*** | 1.00 | 1.00 | 1.00 | 1.00 | 1.00 | 1.00 | 1.00 | 1.00 | 1.00 | 1.00 | 1.00 | 1.00 | 1.00 | 1.00 | 1.00 | 1.00 | 1.00 | 1.00 |
| **0.125** | 0.74 | 0.68 | 0.74 | 0.62 | 0.59 | 0.53 | 0.82 | 0.77 | 0.76 | 0.57 | 0.76 | 0.83 | 0.81 | 0.81 | 0.78 | 0.95 | 0.78 | 0.97 |
| **0.25** | 0.67 | 0.64 | 0.73 | 0.43 | 0.63 | 0.59 | 0.82 | 0.69 | 0.63 | 0.68 | 0.46 | 0.62 | 0.61 | 0.82 | 0.83 | 1.00 | 0.85 | 1.03 |
| **0.5** | 0.63 | 0.61 | 0.74 | 0.49 | 0.49 | 0.45 | 0.75 | 0.61 | 0.59 | 0.65 | 0.45 | 0.65 | 0.56 | 0.70 | 0.79 | 1.03 | 1.11 | 0.87 |
| **1** | 0.52 | 0.68 | 0.57 | 0.42 | 0.37 | 0.49 | 0.74 | 0.57 | 0.58 | 0.62 | 0.43 | 0.50 | 0.47 | 0.50 | 0.42 | 0.92 | 1.03 | 0.95 |
| **2** | 0.51 | 0.57 | 0.49 | 0.37 | 0.43 | 0.53 | 0.69 | 0.54 | 0.53 | 0.50 | 0.43 | 0.75 | 0.47 | 0.44 | 0.41 | 1.08 | 0.98 | 1.24 |
| **4** | 0.53 | 0.45 | 0.49 | 0.36 | 0.50 | 0.31 | 0.52 | 0.43 | 0.47 | 0.36 | 0.47 | 0.59 | 0.38 | 0.48 | 0.33 | 0.92 | 0.96 | 0.97 |
| **8** | 0.51 | 0.41 | 0.35 | 0.31 | 0.39 | 0.28 | 0.42 | 0.44 | 0.41 | 0.47 | 0.24 | 0.51 | 0.41 | 0.22 | 0.33 | 0.97 | 1.05 | 0.92 |
| **16** | 0.27 | 0.28 | 0.21 | 0.29 | 0.27 | 0.11 | 0.27 | 0.27 | 0.33 | 0.24 | 0.23 | 0.38 | 0.34 | 0.31 | 0.27 | 1.08 | 0.95 | 0.82 |

**Table S2.** Extended data for Figure 6 of the main text. 1.00 stands for normalized fluorescence for the untreated cells. Colors of the compounds correspond with the legend of Figure 6.

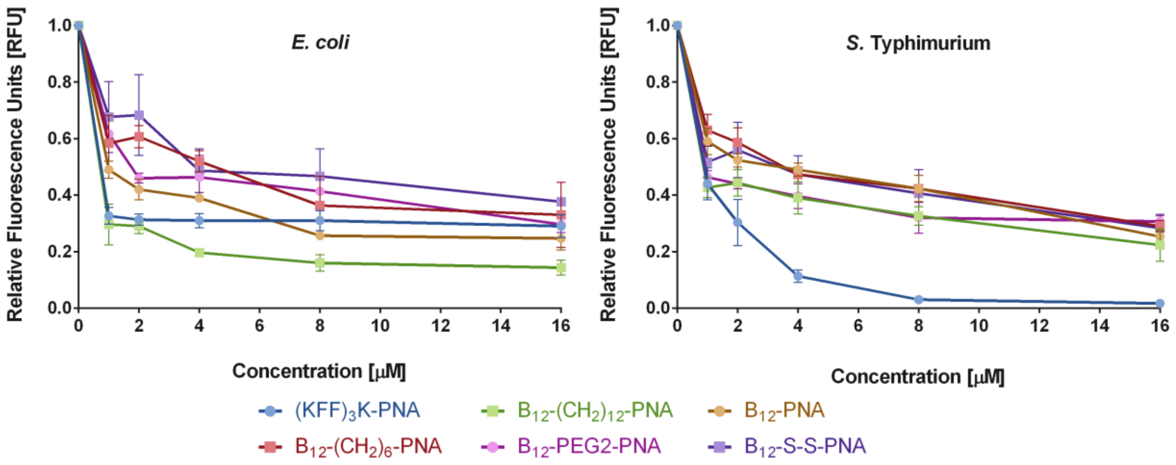


**Figure S2.** Comparison of the effect of anti-*rfp* vitamin $B_{12}$-PNA conjugates and $(KFF)_3K$-PNA on the fluorescence of *E. coli* and *S.* Typhimurium cells. The differences in the fluorescence between *E. coli* cells treated with $(KFF)_3K$-PNA and treated with $B_{12}$-$(CH_2)_6$-PNA, $B_{12}$-PEG2-PNA or $B_{12}$-S-S-PNA are statistically significant ($P \leq 0.01$). In *S.* Typhimurium the differences in fluorescence were significant ($P \leq 0.01$) between $(KFF)_3K$-PNA and all tested vitamin $B_{12}$-PNA conjugates.

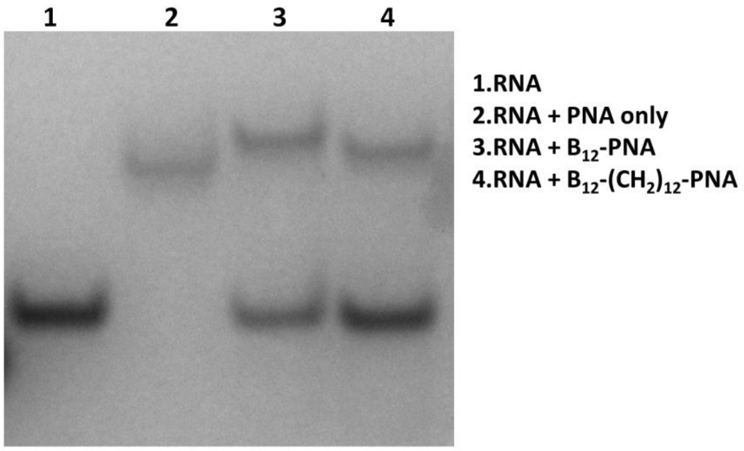


**Figure S3.** The results of polyacrylamide gel electrophoresis experiments in non-denaturing conditions. RNA denotes the mRNA fragment of a sequence $_{5'}$AGGAGAAAUACUAGAUGGCU$_{3'}$ with the region complementary to the 14-mer PNA underlined. RNA was incubated in water solution containing either PNA or the $B_{12}$-PNA and $B_{12}$-$(CH_2)_{12}$-PNA conjugates.

## Section S1: Experimental details and complete characterization of vitamin $B_{12}$ derivatives with aminoazide linkers at 5' position

$^1H$ and $^{13}C$ NMR spectra were recorded at room temperature on a Bruker 500 MHz spectrometer with the residual solvent peak used as an internal standard. Data are reported as follows: chemical shift, peak multiplicity (s = singlet, d = doublet, t = triplet, q = quartet, m = multiplet), coupling constants (Hz), and number of protons. UV–vis spectra were recorded on a Jenway 7315 spectrophotometer. High-resolution ESI mass spectra were recorded on Mariner and SYNAPT spectrometers and on a Q-TOF Premier Waters mass spectrometer

**$B_{12}$-$(CH_2)_{12}$-$N_3$:** red powder; yield: 54%; compound was purified by RP column chromatography gradually with MeCN (acetonitrile) /$H_2O$ (from 15 to 35% v/v). $^1H$ NMR (500 MHz, $CD_3OD$ (deuterated methanol)) δ 7.23 (s, 1H), 7.15 (s, 1H), 6.58 (s, 1H), 6.22 (d, *J* = 2.6 Hz, 1H), 6.04 (s, 1H), 4.64 (dd, *J* = 12.0, 2.1 Hz, 1H), 4.51 (d, *J* = 8.8 Hz, 1H), 4.40 – 4.32 (m, 1H), 4.23 – 4.09 (m, 2H), 4.17 – 4.13 (m, 2H), 3.68 – 3.59 (m, 2H), 3.14 – 3.04 (m, 2H), 2.93 – 2.85 (m, 2H), 2.67 – 2.41 (m, 12H), 2.58 (s, 3H), 2.58 (s, 3H), 2.41 – 2.34 (m, 2H), 2.29 (s, 3H), 2.28 (s, 3H), 2.25 – 2.18 (m, 1H), 2.12 – 1.96 (m, 4H), 1.95 – 1.82 (m, 3H), 1.89(s, 3H), 1.76 – 1.70 (m, 1H), 1.61 – 1.55 (m, 2H), 1.53 – 1.48 (m, 2H), 1.47 (s, 3H), 1.39 (s, 3H), 1.39 –1.37 (m, 2H), 1.37 (s, 3H), 1.36 – 1.27 (m, 16H), 1.25 (d, *J* = 6.2 Hz, 3H), 1.19 (s, 3H), 1.14 – 1.08 (s, 1H), 0.47 (s, 3H). $^{13}C$ NMR (126 MHz, $CD_3OD$) δ 181.6, 180.2, 177.6, 177.4, 177.3, 176.6, 175.5, 175.3, 174.6, 174.1, 167.2, 166.9, 158.7, 143.4, 138.3, 135.7, 133.9, 131.4, 117.9, 112.4, 108.7, 105.2, 95.6, 88.2, 86.4, 81.4, 76.4, 75.2, 73.4, 70.6, 66.9, 64.1, 60.3, 57.7, 56.9, 55.0, 52.6, 52.5, 46.6, 43.9, 43.0, 41.9, 40.4, 36.5, 35.1, 33.2, 33.0, 32.6, 32.4, 32.3, 30.9, 30.7, 30.6, 30.6, 30.5, 30.3, 29.9, 29.5, 27.9, 27.8, 27.4, 27.3, 20.9, 20.5, 20.5, 20.3, 20.2, 20.1, 19.9, 17.5, 17.1, 16.4, 16.1, 15.4. UV/vis ($H_2O$) $\lambda_{max}$ (nm) (ε, L $mol^{-1}$ $cm^{-1}$) 546 ($8.4 \times 10^3$), 519 ($7.5 \times 10^3$), 361 ($2.6 \times 10^3$), 278 ($1.5 \times 10^4$), 221 ($4.6 \times 10^4$). HRMS-ESI (High Resolution Mass Spectrometry Electrospray Ionization) *m/z* $[M + 2Na]^{2+}$ calculated for $C_{76}H_{112}N_{18}O_{15}PCoNa_2$ 826.37043, found 826.37040. Analysis was calculated for $C_{76}H_{112}N_{18}O_{15}PCo{\cdot}5H_2O$: C, 53.77; H, 7.24; N, 14.85. Found: C, 53.67; H, 7.32; N, 15.04. $t_R$ (RP-HPLC, from 10 % MeCN/$H_2O$ + 0.05 % TFA (trifluoroacetic acid) to 70 % MeCN/$H_2O$ + 0.05 % TFA in 15 min and 70 % MeCN/$H_2O$ + 0.05 % TFA in 10 min): 14.50 min.

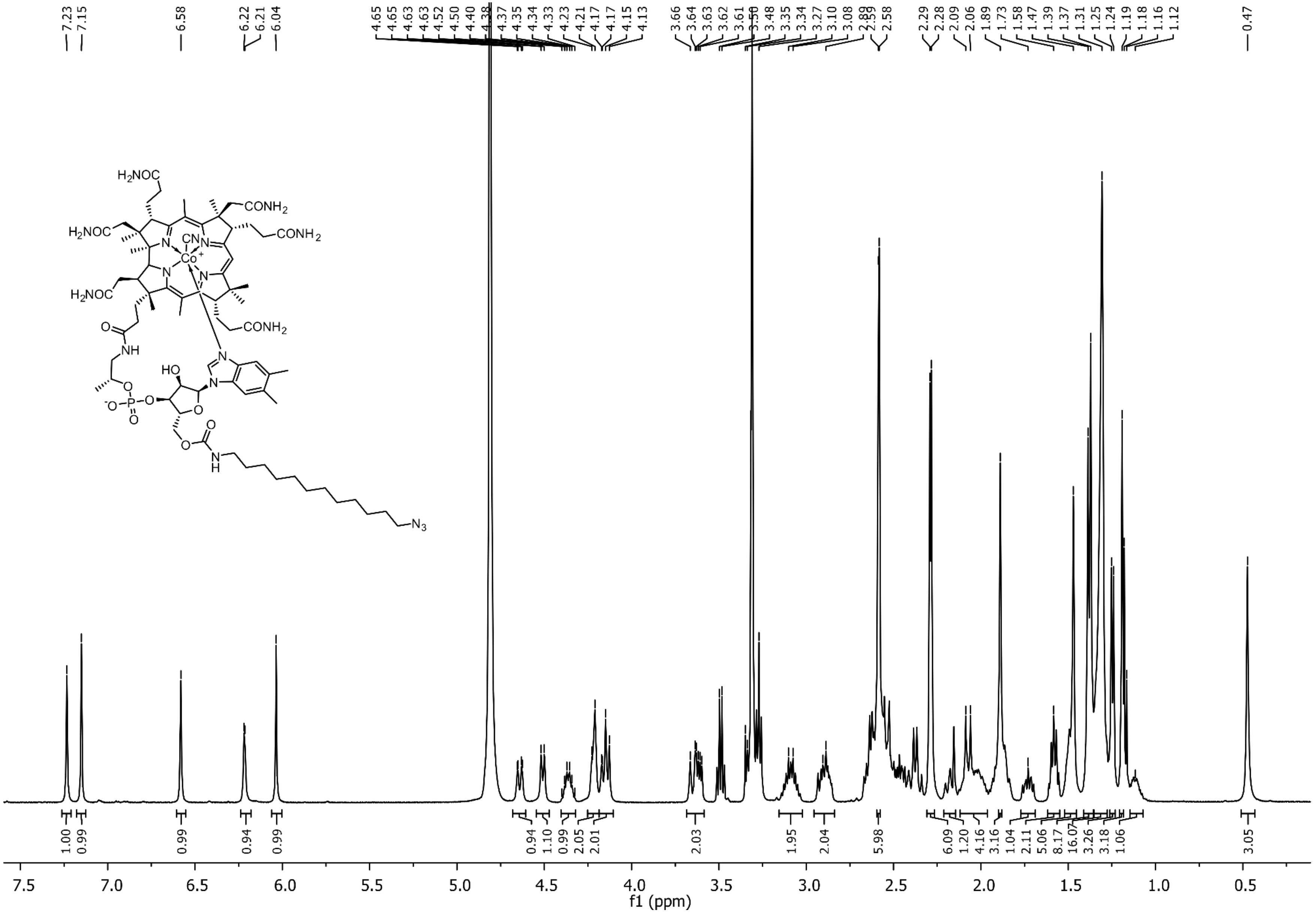
f1 (ppm)

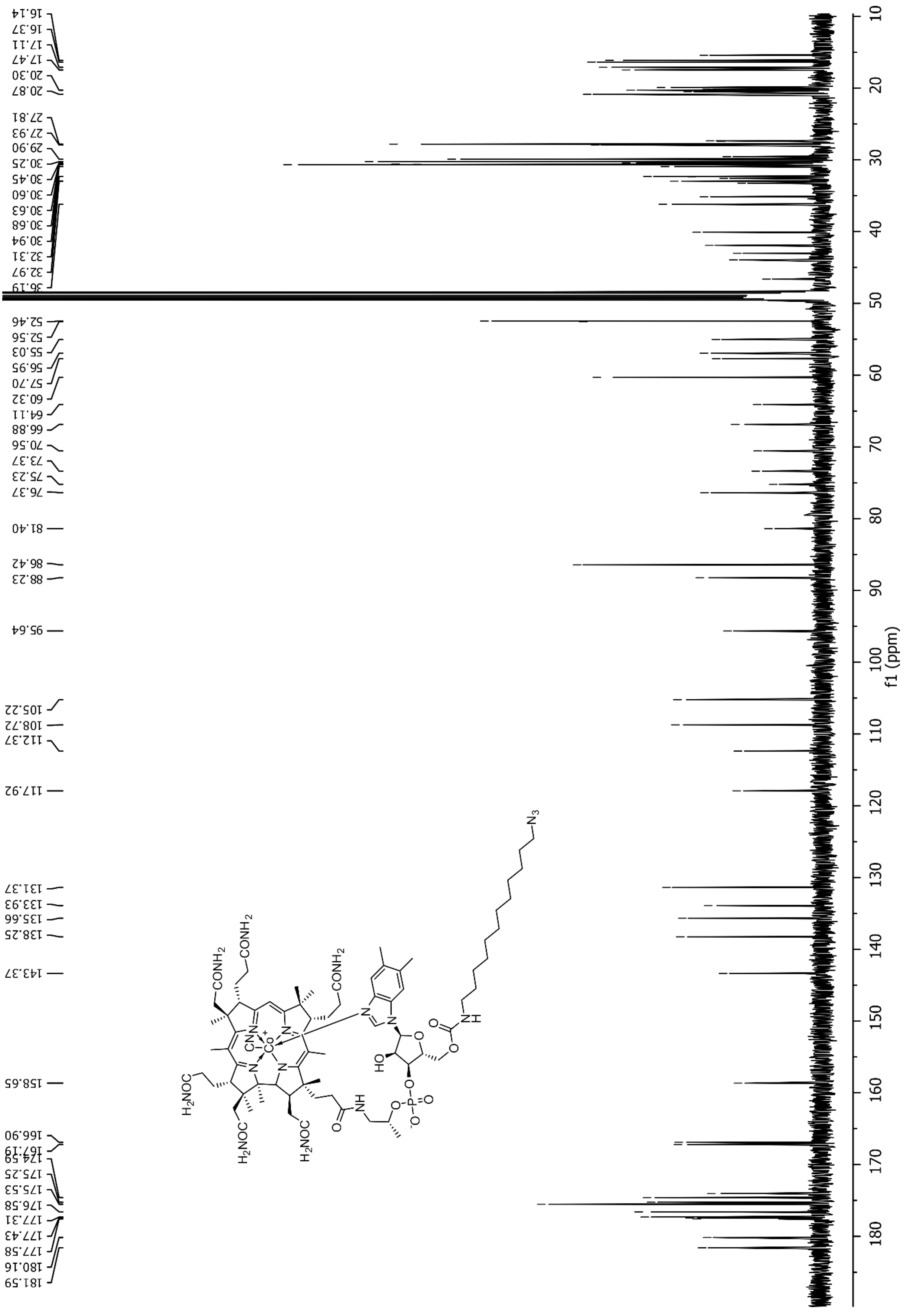


**Figure S4.** NMR spectra of compound $B_{12}$-$(CH_2)_{12}$-$N_3$ recorded in $CD_3OD$ (deuterated methanol)

**$B_{12}$-5'-PEG2-$N_3$**: red powder; yield: 66%; compound was purified by RP column chromatography with MeCN/$H_2O$ (10% v/v). $^1$H NMR (500 MHz, $CD_3OD$) δ 7.25 (s, 1H), 7.15 (s, 1H), 6.58 (s, 1H), 6.23 (d, *J* = 2.6 Hz, 1H), 6.04 (s, 1H) 4.66 (d, *J* = 9.9 Hz, 1H), 4.51 (d, *J* = 8.2 Hz, 1H), 4.40 – 4.32 (m, 1H), 4.24 – 4.20 (m, 2H), 4.17 (dd, J = 12.20, 2.38 Hz, 1H), 4.13 (d, J = 11.50 Hz, 1H), 3.60 – 3.67 (m, 7H), 3.54 (t, J = 5.57 Hz, 2H), 3.36 (t, J = 5.57 Hz, 2H), 2.93 – 2.85 (m, 2H), 2.59 (s, 3H), 2.58 (s, 3H), 2.67 – 2.42 (m, 12H), 2.41 – 2.34 (m, 2H), 2.29 (s, 3H), 2.28 (s, 3H), 2.21 – 2.14 (m, 1H), 2.12 – 1.96 (m, 4H), 1.94 – 1.82 (m, 3H), 1.89 (s, 3H),1.77 – 1.70 (m, 1H), 1.47 (m, 3H), 1.39 (s, 3H), 1.39 –1.37 (m, 2H), 1.37 (s, 3H), 1.30 –1.26 (m 1H),1.25 (d, *J* = 6.27 Hz, 3H), 1.19 (s, 3H), 1.16 – 1.08 (m, 1H), 0.47 (s, 3H). $^{13}$C NMR (126 MHz, $CD_3OD$) δ 180.1, 178.7, 176.1, 176.0, 175.9, 175.1, 174.1, 174.1, 173.8, 173.2, 172.6, 165.7, 165.5, 157.2, 141.9, 136.8, 134.2, 132.5, 129.9, 116.5, 111.0, 107.3, 103.8, 94.2, 86.8, 85.0, 79.9, 74.9, 73.7, 72.0, 72.0, 70.1, 70.0, 69.7, 69.6, 69.1, 62.8, 58.9, 56.2, 55.5, 53.6, 51.1, 50.3, 45.2, 42.5, 41.6, 40.4, 38.7, 34.8, 33.7, 31.8, 31.5, 31.2, 30.9, 30.9, 28.1, 26.0, 25.9, 19.5, 19.1, 19.0, 18.9, 18.7, 18.7, 18.5, 16.1, 15.7, 14.9, 14.7. UV/vis ($H_2O$) $\lambda_{max}$ (nm) (ε, L $mol^{-1}$ $cm^{-1}$) 551 (7.8 × $10^3$), 522 (6.8 × $10^3$), 361 (2.4 × $10^4$), 278 (1.3 × $10^4$), 222 (4.2 × $10^4$). HRMS-ESI *m/z* $[M + Na]^+$ calculated for $C_{70}H_{100}N_{18}O_{17}PCoNa$ 1577.6481, found 1577.6455. Analysis calculated for $C_{70}H_{100}N_{18}O_{17}PCo \cdot 6H_2O$: C, 50.54; H, 6.79; N, 15.15. Found: C, 50.62; H, 7.03; N, 14.95. $t_R$ (RP-HPLC, from 1 % MeCN/$H_2O$ + 0.05 % TFA to 70 % MeCN/$H_2O$ + 0.05 % TFA in 15 min): 10.88 min.

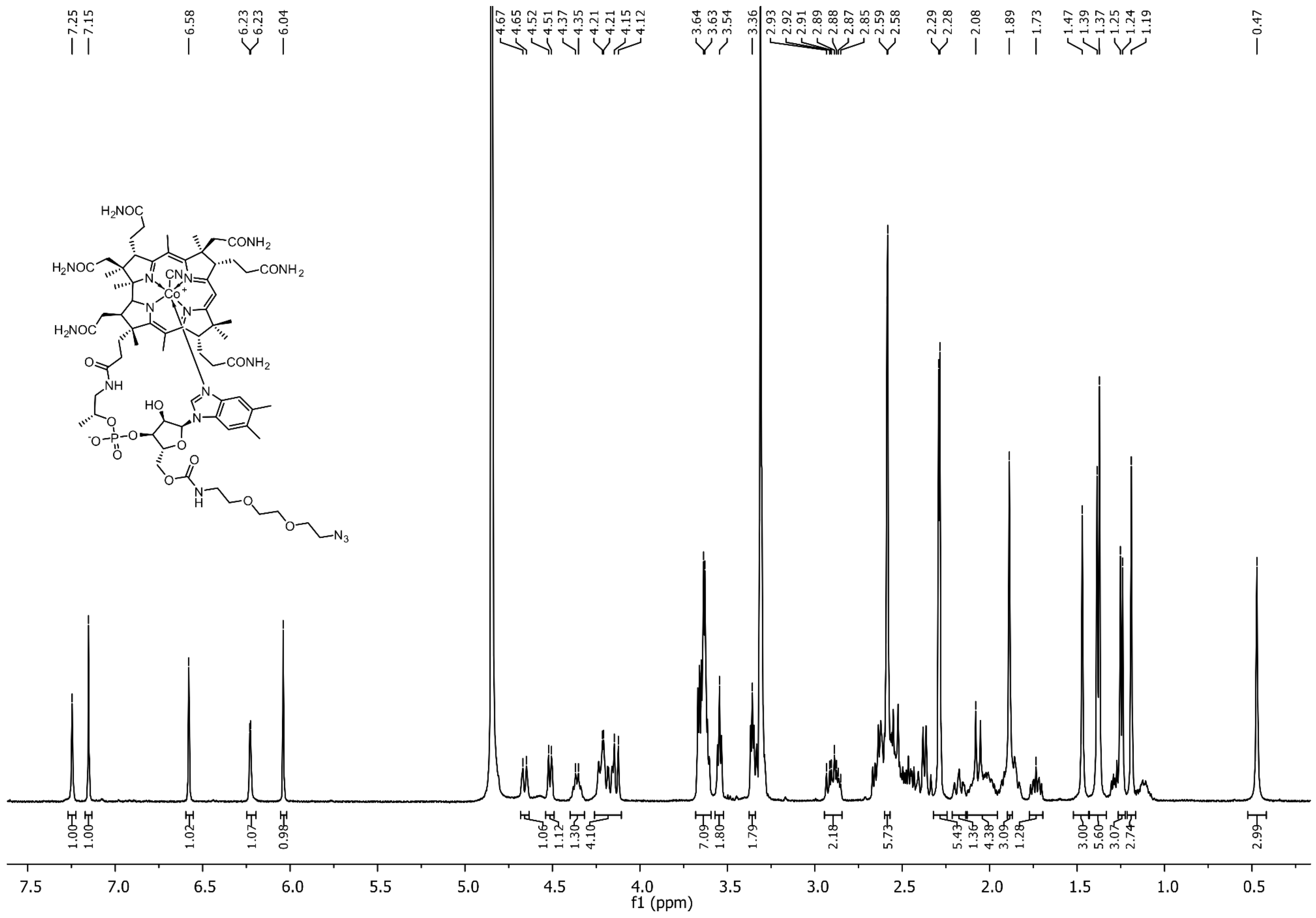

7.25
7.15
6.58
6.23
6.23
6.04
4.67
4.65
4.52
4.51
4.37
4.35
4.21
4.21
4.15
4.12
3.64
3.63
3.54
3.36
2.93
2.92
2.91
2.89
2.88
2.87
2.85
2.59
2.58
2.29
2.28
2.08
1.89
1.73
1.47
1.39
1.37
1.25
1.24
1.19
0.47
1.00
1.00
1.02
1.07
0.98
1.06
1.12
1.30
4.10
7.09
1.80
1.79
2.18
5.73
5.43
1.36
4.38
3.09
1.28
3.00
5.60
3.07
2.74
2.99
7.5
7.0
6.5
6.0
5.5
5.0
4.5
4.0
3.5
3.0
2.5
2.0
1.5
1.0
0.5
f1 (ppm)

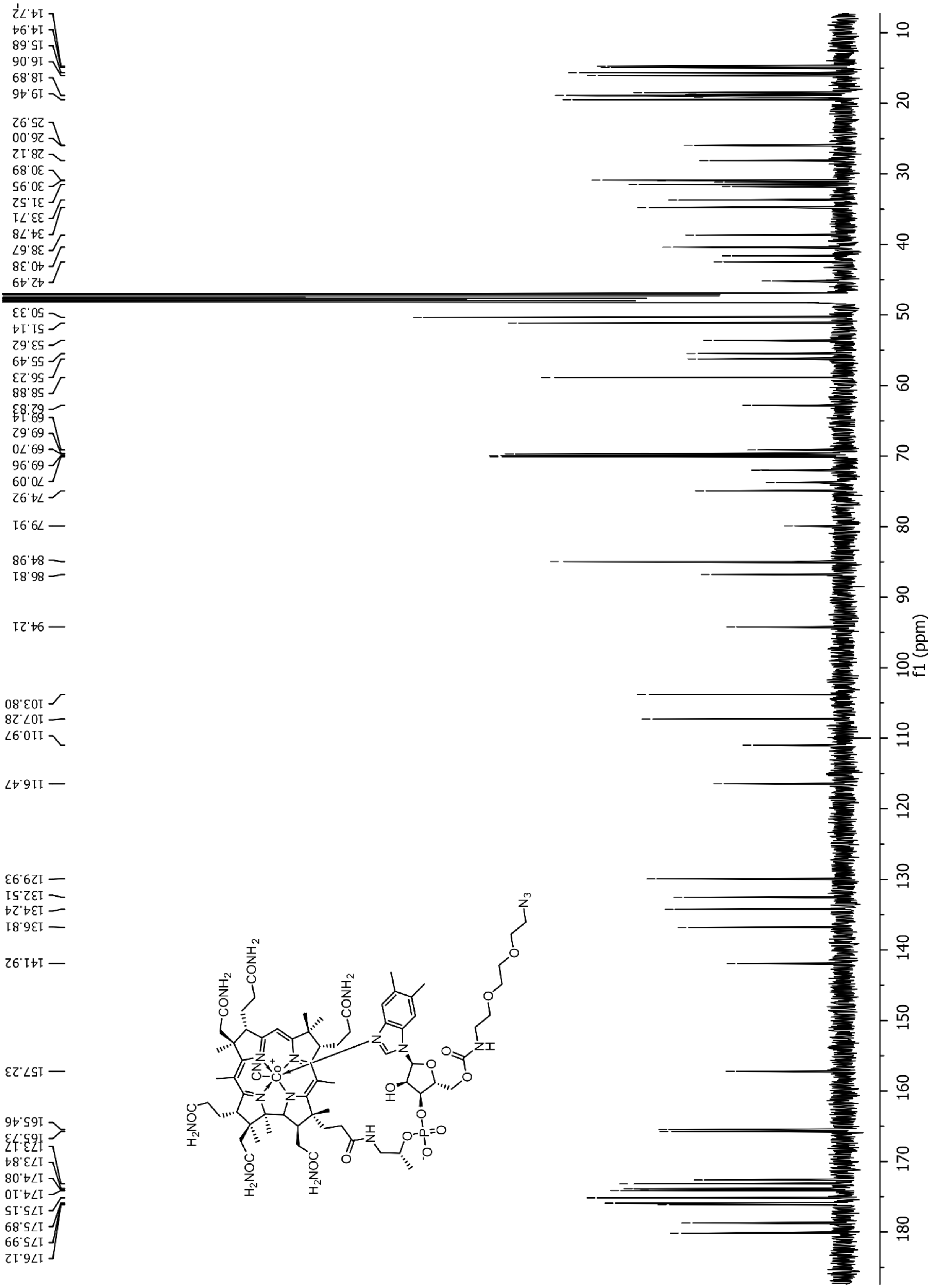


**Figure S5.** NMR spectra of compound $B_{12}$-PEG2-$N_3$ recorded in $CD_3OD$

## Section S2: Mass spectra an RP-HPLC chromatograms of PNA conjugates with vitamin $B_{12}$ and $(KFF)_3K$

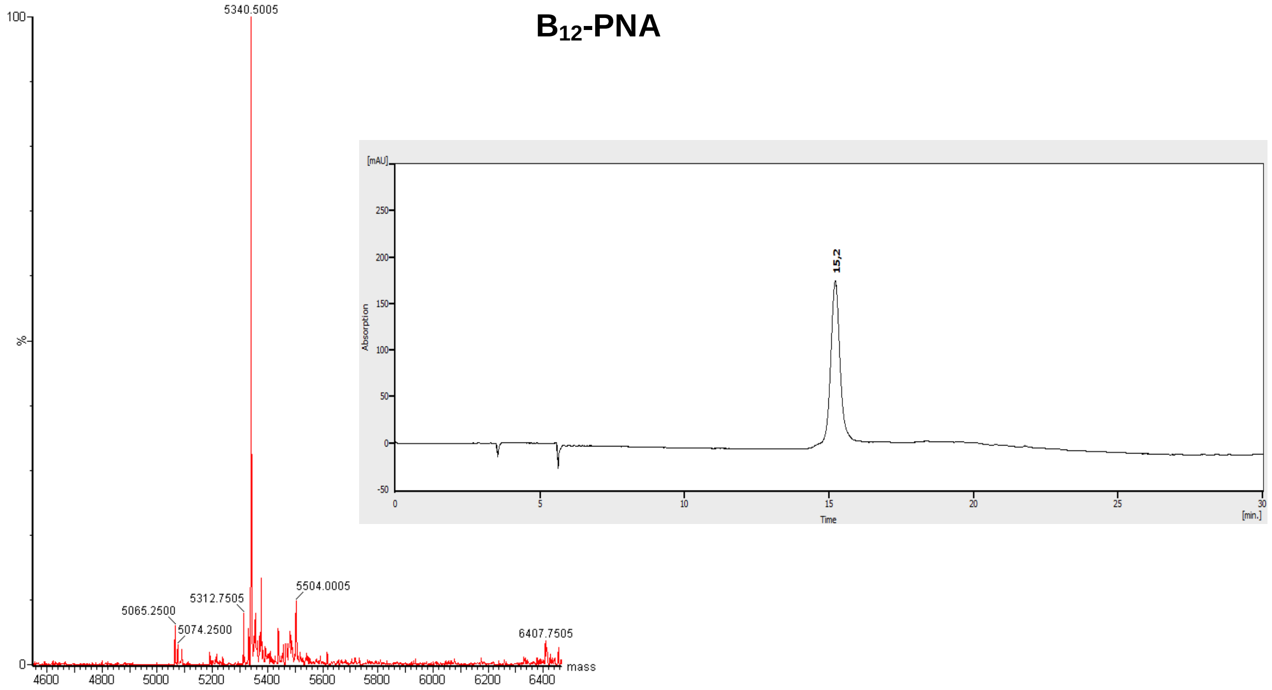


**Figure S6.** Mass spectrum and RP-HPLC chromatogram of $B_{12}$-PNA

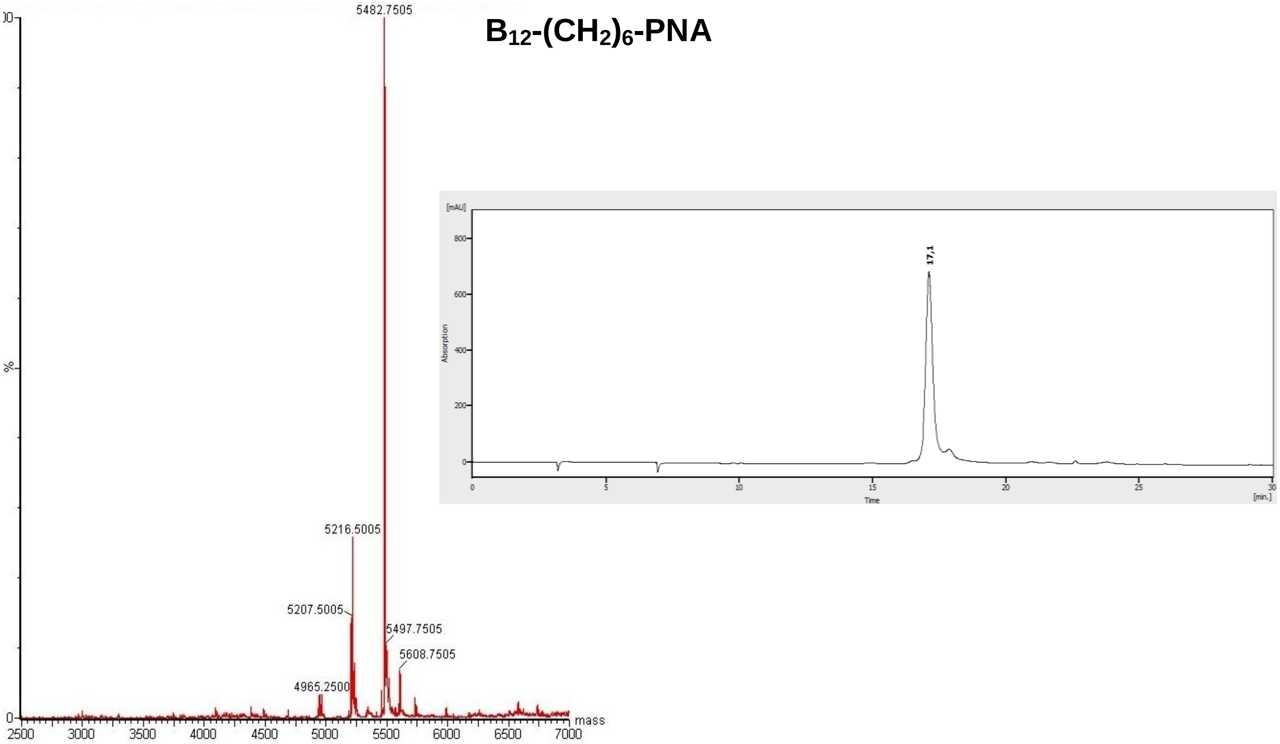


**Figure S7.** Mass spectrum and RP-HPLC chromatogram of $B_{12}$-$(CH_2)_6$-PNA

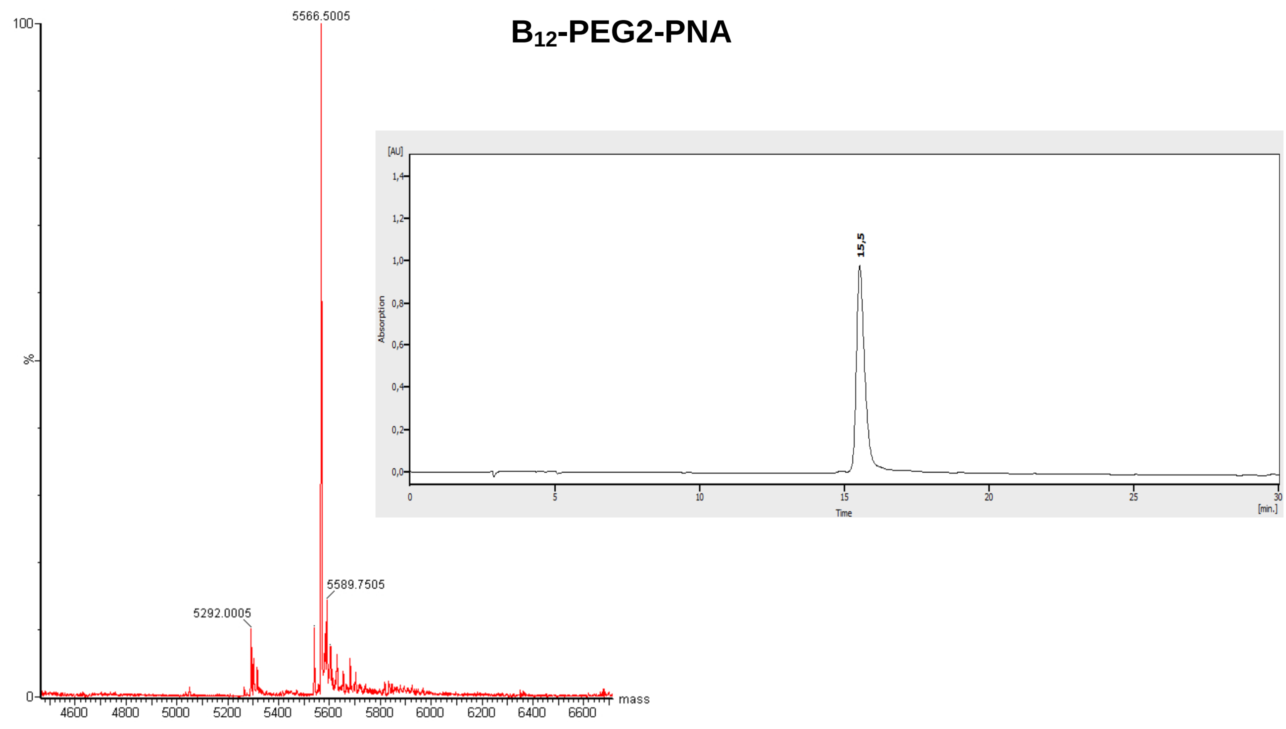


**Figure S8.** Mass spectrum and RP-HPLC chromatogram of B$_{12}$-PEG2-PNA

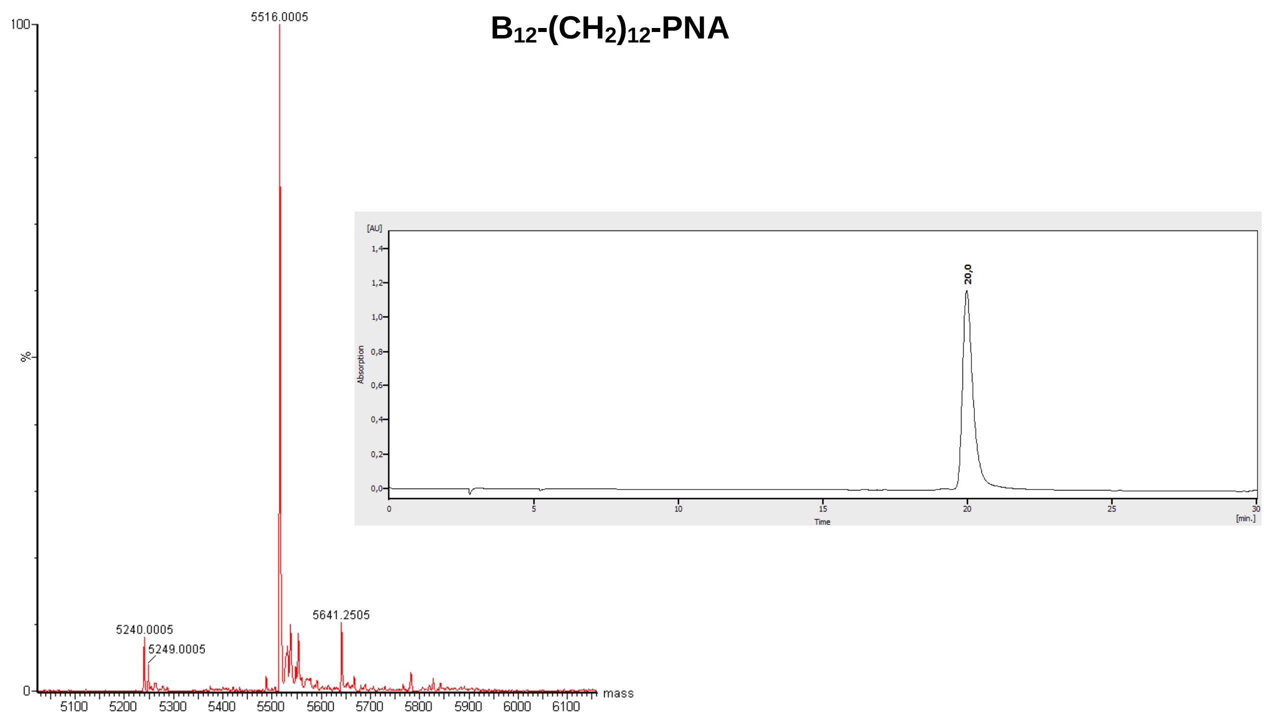


**Figure S9.** Mass spectrum and RP-HPLC chromatogram of B$_{12}$-$(CH_2)_{12}$-PNA

## $B_{12}$-SS-PNA

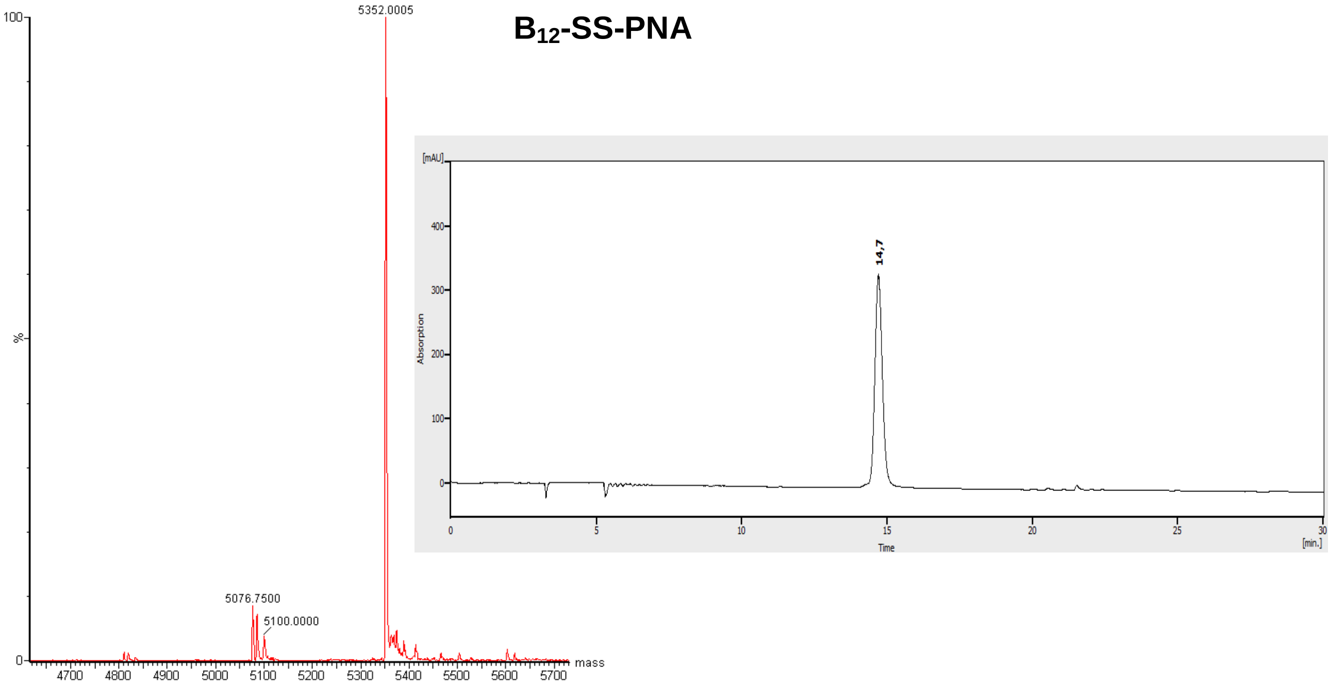


**Figure S10.** Mass spectrum and RP-HPLC chromatogram of $B_{12}$-SS-PNA

## $B_{12}$-$(CH_2)_{12}$-PNA (scrambled)

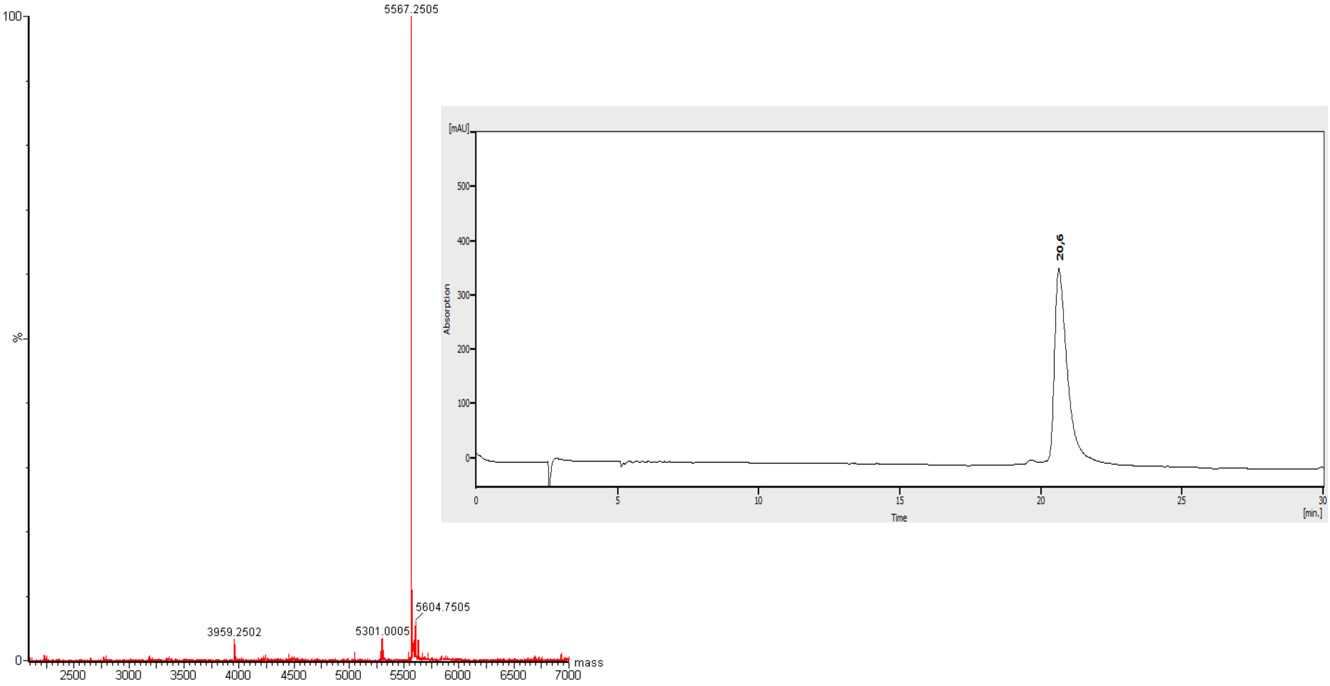


**Figure S11.** Mass spectrum and RP-HPLC chromatogram of $B_{12}$-$(CH_2)_6$-PNA (scrambled)

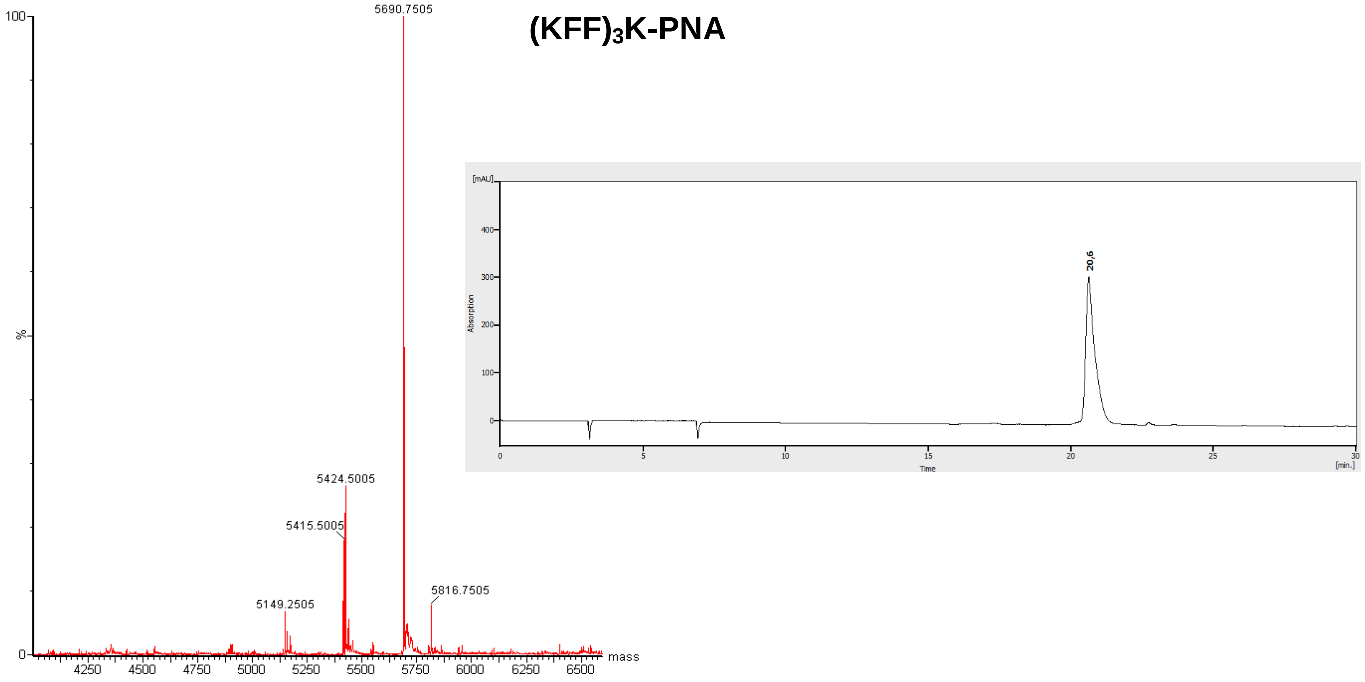


**Figure S12.** Mass spectrum and RP-HPLC chromatogram of $(KFF)_3$K-PNA

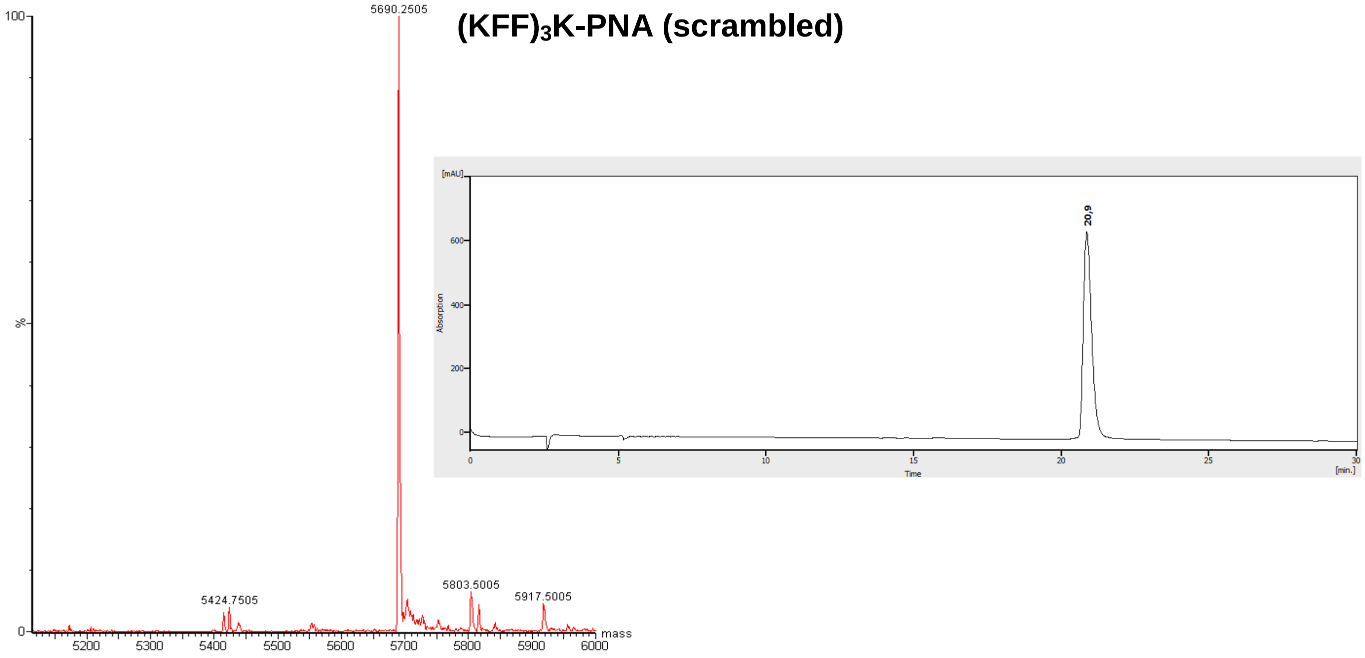


**Figure S13.** Mass spectrum and RP-HPLC chromatogram of $(KFF)_3$K-PNA (scrambled)

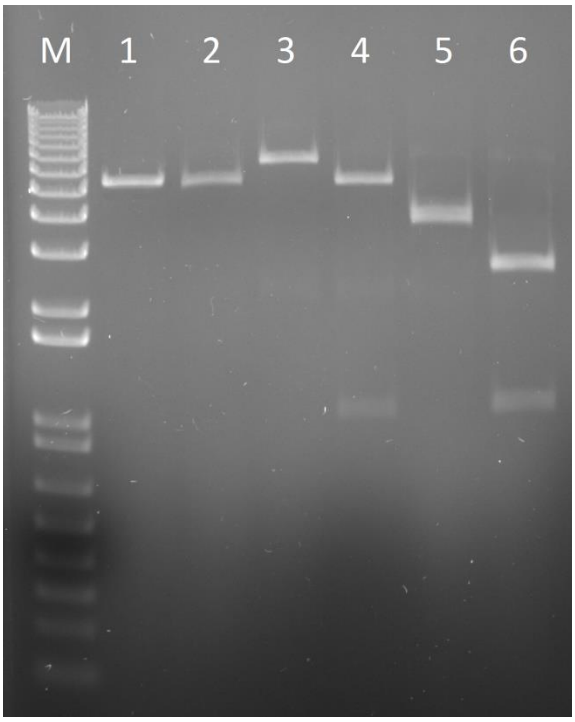


**Figure S14.** Verification of the presence of the pBBR(rfp) plasmid by the restriction digest analysis. M – 1Kb Plus DNA Ladder (Thermo Scientific™, No: 10787018); 1 – pBBR1MCS2 – EcoRI (5144 bp); 2 – pBBR1MCS2 – EcoRI/SpeI (5120 + 24 bp (not visible due to low mass)); 3 – pBBR(rfp) – EcoRI (6212 bp); 4 – pBBR(rfp) – EcoRI/SpeI (5120 + 1092 bp); 5 – pSB3K3 – EcoRI (3819 bp); 6 – pSB3K3 – EcoRI/SpeI (2727 + 1092 bp).

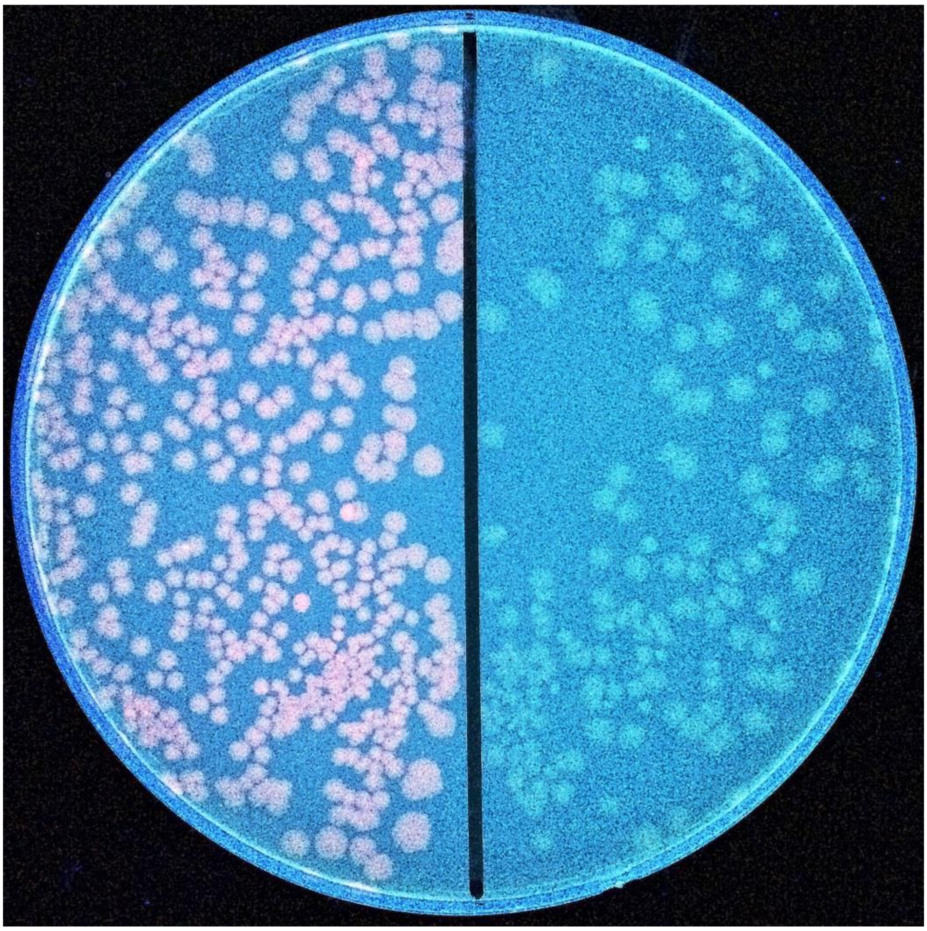

**Figure S15.** The primary detection of recombinants by red – white screening for an altered phenotype caused by the Red Fluorescent Protein (imaged using an UV transilluminator).

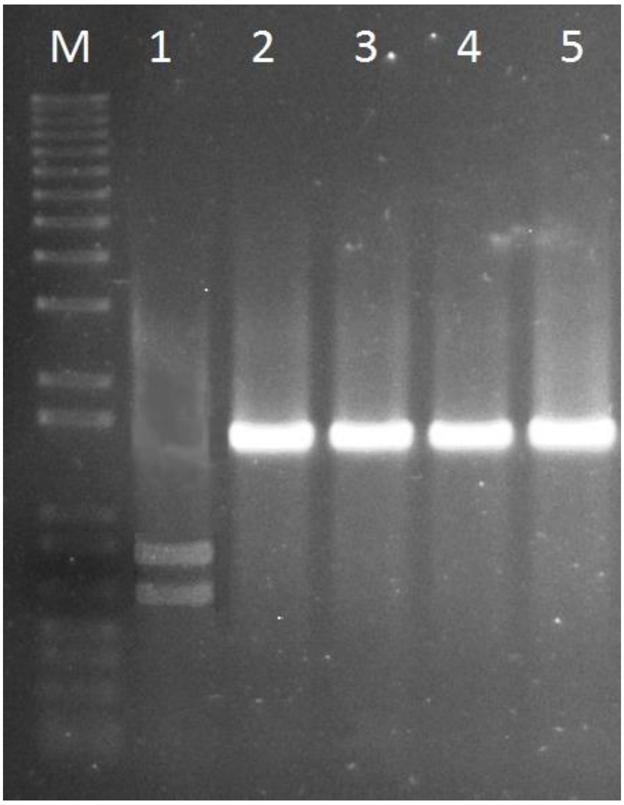


**Figure S16.** Restriction patterns of 16S rRNA genes of *E. coli* (line 1) and *S.* Typhimurium (lanes 2-5) digested with SalI. Lane M, size marker 1Kb Plus DNA Ladder (Thermo Scientific™, No: 10787018). Restriction enzyme SalI does not digest *S.* Typhimurium 16S rRNA (1520 bp); however 16S rRNA of *E. coli* was digested in 3 parts (1542 bp = 53*, 668, 821 bp) where * stands for an invisible band due to low molecular weight.
